# Acceptability of community-based mentor mothers to support HIV-positive pregnant women on antiretroviral treatment in western Kenya: a qualitative study

**DOI:** 10.1186/s12884-019-2419-z

**Published:** 2019-08-13

**Authors:** Iris Wanga, Anna Helova, Lisa L. Abuogi, Elizabeth A. Bukusi, Wafula Nalwa, Eliud Akama, Thomas A. Odeny, Janet M. Turan, Maricianah Onono

**Affiliations:** 1 Centre for Microbiology Research, Kenya Medical Research Institute, P.O. Box 19464 - 00202, Nairobi, Kenya; 2 Department of Health Care Policy and Organization, School of Public Health, University of Alabama at Birmingham, 517 RPHB 1665 University Blvd, Birmingham, AL 35294 USA; 3 Department of Pediatrics, University of Colorado Denver, 13199 East Montview Blvd, Suite 310 Mail Stop A090, Aurora, CO 80045 USA; 4 Maseno University School of Medicine, Box 3365-40100, Kisumu, Kenya

**Keywords:** Prevention of mother-to-child transmission, Community mentor mothers, HIV, Option B+

## Abstract

**Background:**

Option B+ is a comprehensive antiretroviral treatment (ART) designed for HIV-infected pregnant/ postpartum women. However, barriers to implementing Option B+ and establishing long-term ART adherence while facilitating retention in prevention of mother to child transmission of HIV (PMTCT) services remain. Community-based mentor mothers (cMMs) who can provide home-based support for PMTCT services may address some of the barriers to successful adoption and retention in Option B+. Thus, we evaluated the acceptability of using cMMs as home-based support for PMTCT services.

**Methods:**

Gender-matched in-depth interviews were conducted between September–November 2014 for HIV-infected pregnant/postpartum women and their male partners living in southwestern Kenya (*n* = 40); additionally, we conducted four focus groups involving 30 health workers (*n* = 70) within four health facilities. Audio-recordings were transcribed, translated, and then coded using a thematic analytical approach in which data were deductively and inductively coded with support from prior literature, identified themes within the interview guides, and emerging themes from the transcripts utilizing Dedoose software.

**Results:**

Overall, the study results suggest high acceptability of cMMs among individual participants and health workers. Stigma reduction, improvement of utilization of health care services, as well as ART adherence were most frequently discussed potential benefits of cMMs. Participants pictured a cMM as someone acting as a role model and confidant, and who was over 30 years old. Many respondents raised concerns about breaches of confidentiality and inadvertent disclosure. Respondent suggestions to overcome these issues included the cMM working in different communities than where she lives and attending home-visits with no identifying clothing as an HIV-related health worker.

**Conclusions:**

The home-based cMM approach may be a beneficial and acceptable strategy for promoting ART adherence and retention within PMTCT services for pregnant/postpartum women living with HIV. Considering the risks of inadvertent disclosure of HIV-infected status and related negative consequences for pregnant/postpartum women living with HIV, similar cMM program designs may benefit from recognizing and addressing these risks.

**Trial registration:**

The MOTIVATE! study was registered on July 7, 2015 at the ClinicalTrials.gov (NCT02491177).

**Electronic supplementary material:**

The online version of this article (10.1186/s12884-019-2419-z) contains supplementary material, which is available to authorized users.

## Background

Despite considerable progress in the prevention of new HIV infections, 180,000 new pediatric infections were reported in 2017 primarily due to perinatal and postpartum mother to child transmission of HIV [1, 2]. The majority of these new pediatric infections occurred in sub-Saharan Africa (SSA) where nearly 990,000 women were HIV-infected during pregnancy in 2016 in the 21 Global Plan priority countries [1, 3]. The proportion of women living with HIV accessing antiretroviral treatment (ART) in these priority countries has increased from 36% in 2009 to 80% in 2017 [2] which resulted in a global reduction of the mother-to-child transmission (MTCT) rates from 22 to 11% [1, 4]. However, additional efforts including universal HIV testing and counseling for pregnant and breastfeeding women, high coverage with effective PMTCT/ART regimens, and support for adherence are needed to achieve the Global Plan goals [2–4].

According to 2016 World Health Organization guidelines, comprehensive ART treatment is recommended for all pregnant or breastfeeding women to be initiated immediately after diagnosis irrespective of CD4 count or clinical stage of disease (known as Option B+). Infants receive daily nevirapine or azidothymidine from birth until 4–6 weeks after birth regardless of the feeding method [3, 5, 6]. However, it is clear that scale-up of ART and Option B+ will increase the burden on already overwhelmed health care facilities and current workforce [7]. The United Nations’ established the “Start Free. Stay Free. AIDS Free.” and the Sustainable Goal Development framework to further the Global Plan for eliminating new infant and child HIV infections [1, 2, 8]. UNAIDS recommends decentralization of services to the lowest levels, mobilization of affected communities in service provision, and female empowerment [9, 10]. Existing research suggests that community engagement, especially involvement of people living with HIV, is critical for PMTCT and in particular, can improve retention in the PMTCT cascade [11–15].

Mentor mothers exemplify the UNAIDS recommended model by utilizing task shifting, community engagement, as well as female empowerment of PLHIV. The original Mother2mothers (m2 m) program was established in South Africa in 2001 and implemented in 10 countries in SSA, including Kenya. A mentor mother is an HIV-infected mother trained and employed by a health care facility [3, 16], who is responsible for giving one-on-one support to HIV-infected pregnant/postpartum women; encourage enrollment, adherence and retention in HIV care, perform tracing for women who miss clinic visits; and educate on PMTCT and health-related topics [16, 17]. In 2017, m2 m program facility-based mentor mothers have assisted nearly 2.5 million women living with HIV in SSA [18]. Programs in countries with mentor mothers such as Uganda, South Africa, Nigeria, Ethiopia, and Malawi report lower MTCT rates, improved ART adherence, increased uptake of early infant HIV diagnosis, and reduced workload on skilled health workers [19–27]. However, rigorous study of the impact of mentor mothers has mostly been reported for mentor mothers working and supporting women in health facilities or with very limited community-based activities.

Despite being listed as a priority country for addressing new child and infant HIV infections, Kenya has an estimated 6,500 (2016) new pediatric HIV infections annually [1, 10]. The Kenyan government formalized the roll-out of Option B+ in 2013, and is credited with an increase from 52% in 2009 to 80% in 2016 in the number of pregnant women who are HIV-infected and are receiving life-long ART [1, 17, 28]. However, challenges remain that may limit the scale-up of ART services and Option B+. The Kenya Mentor Mother Program (KMMP) was established in 2012 [29]. In 2014, 325 facility-based mentor mothers whose salaries were supported by implementing partner organizations were working at 236 health care sites in Kenya [16].

Building on the success of facility-based MMs in SSA settings [21, 24], our team proposed the implementation of a community-based mentor mother program. We used qualitative methods to explore the feasibility and acceptability of community-based mentor mothers to potentially improve ART adherence and retention in HIV care with respect to the comprehensive approach of Option B+.

## Methods

Formative data were collected between September and November 2014. Findings from this study were used to refine the data collection instruments and intervention plans in the quantitative stages of the MOTIVATE! trial (Mother and Infant Visit Adherence and Treatment Engagement) [30] initiated in December 2015. The MOTIVATE! trial is evaluating the impact of cMM and text messaging interventions to support comprehensive ART adherence and PMTCT retention for Option B+ implementation in Kenya using a 2 × 2 factorial design (Clinicaltrials.gov Identifier: NCT02491177). Eligibility criteria for the randomized trial following the formative qualitative part of the study included HIV-infected pregnant woman 18 years of age or older, having access to a mobile phone, and agreeing to cMM visitation and residing within the catchment area served by the health facility where they enrolled in the study. Details of the MOTIVATE! study protocol and intervention design have been published elsewhere [30].

### Setting

This formative study was conducted in four health facilities located in Homa Bay, Migori and Kisumu Counties of southwestern Kenya. Approximately a third of all HIV infections in Kenya are located in this region [31, 32]. HIV county prevalence rate remains high: 14.3% in Migori, 19.9% in Kisumu, and 26.0% in Homa Bay County [33]. In 2013, about 15,000 women in these counties received maternal prophylaxis for PMTCT with estimated 62–88% coverage [34]. The main economic activities in these three counties include fishing and subsistence farming.

### Proposed intervention-community Mentor mothers

This cMM strategy was developed by the MOTIVATE! study team [30]. Similar to facility-based mentor mothers, cMMs are HIV-infected women with recent (6 months to 2 years) pregnancy experience and on Option B+ ART. They serve as peer mentors to HIV-positive pregnant and postpartum women and aim to improve PMTCT service uptake and retention. Table 1 outlines differences between cMMs and facility-based mentor mothers. The cMMs receive a two-week intensive training based on curricula adapted from UNICEF, Medicine Sans Frontiers, and KMMP [29].

**Table 1:** Comparisons between facility-based and community-based mentor mothers

	Facility based MM	Community MM
Remuneration and workload		
Receive stipend	✓	✓
Receive other benefits e.g. health insurance	✓	✓
Conduct support groups at the facility	✓	
Facility-based client interactions	✓	✓
Community-based client interactions		✓
Involved in facility register documentation	✓	
Keeps record/logbook of peer support activities	✓	✓
Core adherence and retention activities		
Phone call/SMS	✓	✓
Adherence/retention counseling	✓	✓
Regular home/non-facility visits during pregnancy and postpartum		✓
Liaise with or alert health care workers for tracking clients	✓	✓
Support to male partners		✓
Support to children/family		✓

The cMMs engage with HIV-infected pregnant woman at earliest identification within the antenatal clinic and follow these women up to at least 1 year postpartum. Follow-up involves up to 4 prenatal and 9 postnatal home visits tailored to complement pre- and postnatal antenatal care visits, infant immunization schedules, and infant HIV testing in line with the Kenyan guidelines for caring for the newborn at home [35]. These guidelines advocate for five visits with 2 prenatal visits occurring 4 weeks apart, 1 delivery day visit and 2 postnatal visits one on day 3 and day 7 after delivery. In order to reinforce information or health topics discussed by the service provider at the health facility, we added home visits at 6, 10, and 14 weeks of infant age, and at months of age 6, 9, and 12 which coincide with immunization dates and important dates for early infant diagnosis and testing and follow up. The topics discussed during home visits include: promotion of antenatal care (ANC), birth planning, adherence to antiretroviral drugs, immediate newborn care, family planning, care of the HIV exposed infant (including infant prophylaxis and early infant diagnosis), feeding methods and weaning. The cMMs collect data at each visit on mobile phones which have a data collection software (Open Data Kit) installed on them. This application is used not only to collect data, but also prompts the cMMs to cover specific health topics. The health topics to be discussed at each visit are dependent on the gestation of the pregnancy or the age of the infant.

### Data collection methods and selection of study participants

As this was a formative study investigating the feasibility and acceptability of community-based mentor mothers, we adapted a thematic analysis approach, and grounded our findings in the data rather than using an existing theoretical framework. A convenience sample of participants (*n* = 70) meeting eligibility criteria set for the qualitative phase of the MOTIVATE! Trial participated in in-depth interviews (IDI) between September and November 2014. Women were eligible if they were HIV-infected and pregnant/postpartum, 18 years or older and willing to participate. Male partners of HIV-infected pregnant woman were eligible if they were aware of partners’ HIV status, 18 years or older and willing to participate. Health care workers currently employed at one of the 20 study sites or part of the county health management team were considered to be eligible. Reporting of this study adheres to the consolidated criteria for reporting qualitative research (COREQ) guidelines.

#### In-depth interviews

Gender-matched in-depth interviews were conducted involving 40 HIV-infected pregnant women and those within 6-weeks-postpartum (*N* = 20), along with their male partners (*N* = 20), half of which were HIV-infected living in seroconcordant relationships and half which were HIV-negative living in serodiscordant relationships. Health workers, registers, and patient files at health care facilities providing Option B+ services were consulted and used to identify potential participants. A short private session was used to evaluate interests in study participation and to identify eligibility among potential participants, plain-language explanations were provided regarding the separation of study participation from regular medical care and the option to decline to participate in the study. Male partners were contacted regarding potential participation in an interview if female participant agreed to it and has previously disclosed her HIV status to her male partner. Private settings were used for conducting the individual interviews offered in English or Dholuo language, according to the participant’s preference, and lasted approximately 1–1.5 h. Demographics, employment, pregnancy and HIV-related information were collected for each participant.

#### Focus groups

Various health care providers (*n* = 30) were purposively selected for inclusion in the focus groups (7–8 from each of the four selected facilities). These included facility nurses, community health workers, health educators, mentor mothers, counselors for HIV, laboratory technicians, facility in-charges, program technical advisors, and administrative staff all selected for maximum variation in occupational perspectives. The research coordinator invited potential participants to focus groups at their health facility. Characteristics of participants were collected, including demographics (gender, age, educational level, marital status, the number of living children, clinic name) and employment characteristics (length of time in the current employment, current employment description). Focus groups lasted approximately 2–3 h.

Guides for in-depth interviews and the focus group discussions were based on published literature and the research team’s prior experience and research regarding pregnancy and HIV in similar settings [36, 37]. Topics developed within the two guides pertained to the acceptability of comprehensive ART for pregnant HIV-infected women, barriers and facilitators to HIV-care adherence and retention, and the acceptability of the cMM approach. English and Dholuo interview and focus group discussion guides are available as Additional files 1 and 2 respectively. This manuscript focuses on findings on the cMM approach. Findings pertaining to challenges in the health facilities on providing Option B+ are published elsewhere [38]. One male and one female experienced interviewers who were fluent in English and Dholuo, completed training in both qualitative research methods and the study topics.

### Data management and analysis

Digital audio- recordings were collected for the in-depth interviews and focus groups and then stored under password-protection in a secure location with regards to the participants’ privacy. These recordings were translated into English if necessary, and transcribed verbatim by trained qualitative staff with identifying data removed. Interviewers/moderators took notes for purposes of assistance with transcription. Dedoose qualitative software program (Sociocultural Research Consultants, LLC) was utilized, with coding and data analysis following a thematic analytic approach [39, 40]. Thematic analysis was chosen as our method of choice owing to its flexibility as it is characteristically independent of theory and epistemology [41]. A framework for qualitative coding was developed using existing literature, interview guides, and an emergent theme approach with data derived from the transcripts. A multi-step inductive process of coding was used in which transcripts were initially broadly-coded by three trained qualitative coders who then followed with fine-coding allowing for the emergence of additional topics, the refinement of major themes and identification of sub-themes. The coders established consistency of coding beginning by coding the same transcripts together and with frequent discussions between coders throughout the process. Data saturation was discussed and jointly concluded that the data saturation was reached. Findings were disseminated at participating health facilities and communities.

## Results

### Participant characteristics

Table 2 presents the participants’ sociodemographic characteristics. Seventy participants were involved in the in-depth-interviews and focus groups comprised of 20 pregnant/ postpartum women with HIV-infection (mean age 24.7 years ±4.8) and their male partners (mean age 33.5 years ±8.8), and a total of 30 health care providers (mean age 32.2 years ±7.2). More than 80% of participants were married, a majority were living with their spouse (93%), and about two-thirds had at least 2 living children (66%). Of the female and male partners participants, the majority had not attained more than primary education (70%). However, all health care providers in the focus groups completed at least secondary education, and a majority worked 3 or more years in their current profession in health/community services (67%). Recruitment in the study was designed to incorporate participants such that half of the individual interview participants were living in HIV-seroconcordant relationships and half were living in HIV-serodiscordant relationships. The results were organized into four thematic areas: overall acceptability, ideal roles, ideal characteristics, and potential risks of the cMM program (see Table 3).

**Table 2:** Socio-demographic and HIV-related characteristics

Characteristics	Female participants *N* = 20	Male participants *N* = 20	Health workers *N* = 30
Age: Mean (SD)	24.7 (4.8)	33.5 (8.8)	32.2 (7.2)
Participant education: N (%)			
Did not complete primary	11 (55.0)	6 (30.0)	0 (0)
Complete primary	5 (25.0)	6 (30.0)	0 (0)
Did not complete secondary	1 (5.0)	3 (15.0)	0 (0)
Complete secondary	3 (15.0)	5 (25.0)	10 (33.3)
Any college	0 (0)	0 (0)	20 (66.7)
Marital status: N (%)			
Monogamous marriage	16 (80.0)	18 (90.0)	20 (66.7)^a^
Polygamous marriage	3 (15.0)	2 (10.0)	
Single	1 (5.0)	0 (0)	8 (26.7)
Widowed	0 (0)	0 (0)	2 (6.7)
Current occupation: N (%)			
Agriculture	3 (15.0)	9 (45.0)	0 (0)
Business/sales	5 (25.0)	2 (10.0)	0 (0)
Health/community services	0 (0)	0 (0)	30 (100.0)
Skilled worker	2 (10.0)	9 (45.0)	0 (0)
Housework	9 (45.0)	0 (0)	0 (0)
None	1 (5.0)	0 (0)	0 (0)
Length of time in current occupation: Mean (SD)	Not asked	Not asked	4.7 (5.1)
Number of Living Children: Mean (SD)	2.2 (1.1)	3.2 (1.9)	1.8 (1.8)

^a^Married. Information on type of marriage not collected for health workers

**Table 3:** Summary of themes related to potential feasibility and acceptability of a cMM program in southwestern Kenya

Themes	Key results
Perceived benefits and acceptability of cMMs	• Advantage of having a woman directly in the community with recent PMTCT experience
	• Potential for HIV-related stigma reduction
	• Female empowerment
	• Reduction of healthcare providers’ workload
	• Elimination of travel to clinic for certain services
Ideal role of cMMs and ideal aspects of cMM visit	• Target population: HIV-infected pregnant or postpartum women, extended groups (people at HIV risk)
	• Topics to be covered during the visits: PMTCT, reproductive health, tools to overcome fears related to living with HIV, child health, first aid, marital counseling, non-communicable diseases, TB, other
	• Private visits, male partner involvement in at least part of the visit
	• No universally convenient timing for the visits – to be adjusted to individual /community preferences
Ideal characteristics of cMMs	• HIV-infected and open about her HIV status
	• Good adherence to HIV treatment
	• Familiar with community culture and beliefs
	• Ability to maintain strict confidentiality
	• Middle-aged
	• Knowledgeable and a good educator
	• Good communication skills
	• Friendly and engaging
Potential risks and barriers related to the cMM approach	• Breach of confidentiality
	• Inadvertent disclosure of HIV status
	• Lack of private meeting places in clients’ homes
	• Potential stigmatization and social isolation of cMMs in the communities

#### Theme 1: overall acceptability of community-based Mentor mothers

A majority of the interviewed women, their male partners, and health care providers indicated overall acceptability of the planned cMM intervention. The participants expressed that there is an advantage of having a woman from their community with recent PMTCT experience providing services and encouragement to pregnant/postpartum women.

*“It is good because the mentor is from the community and somebody known to you personally and understands you”. (HIV-infected, Male, 35–39 years)*

*“It can be great and also of benefit to many. Because people are afraid of speaking openly about their HIV status and now they will find somebody who will help them open up and encourage them to go to the hospital. That can be of benefit to several people”. (HIV-infected, female, 20–24 years)*

A major benefit of cMMs identified by participants was the potential for cMMs to help reduce HIV-related stigma in the community, allowing people to speak more freely about their HIV status. *“That can help because when they work in the community they remove fear from people, give people positive thoughts, show people how to work and live together making people to be free”. (Health worker, Male, 35–39 years)*

Another benefit discussed by the participants was the potential to empower both pregnant women living with HIV and the mentor mothers themselves.

*“…the program is going to empower women around. You know when you empower a person you will change the life of this person and she will see that HIV is not a disability to her and she will have something at the end of the month because you cannot have a mentor mother without salary. So she will be taking it upon herself to be doing her work perfectly”. (Health worker, female, 30–34 years)*

Additionally, clinic staff felt cMMs might reduce healthcare providers’ workload through task shifting.

*“That program of mentor mother in the community will help a lot because we are experiencing understaffing, so moving to the community to trace client is a bit technical. Mentor will help us in the tracing of defaulter”. (Health worker, female, 40–44 years)*

There were diverse views of where cMMs should be based. Some participants expressed belief that having a resident cMM would eliminate the need for mothers to travel to a clinic or be seen visiting the HIV clinic.

*“That would be a very good intervention because when they conduct home visits, they will be able to educate the mothers and other people won’t know what they discussed. The mothers therefore won’t necessarily need to be in the clinic to learn or get educated”. (HIV-infected, Male, 30–34 years)*

#### Theme 2: ideal role of a community mentor mother

Most participants preferred cMMs to visit only HIV-infected pregnant or postpartum women and to focus their education and support on HIV and PMTCT. Other participants suggested targeting additional groups (all pregnant women, male partners, neighbors, and all sick people) and including broader topics in the visits. Reasons given for targeting these additional groups included raising awareness of HIV and PMTCT among all groups at risk, to avoid unintentional disclosure of HIV status that might occur by focusing only on women living with HIV, and to act as a link between the larger community and the health facilities.

*“She should visit ALL women in the community. A woman might be pregnant for close to 5 months and has never stepped into a clinic and she might also be HIV-infected , yet she is not aware. The mentor mother will encourage her to go to the clinic the next day; she will be tested for HIV and if the result will be positive, she will immediately be enrolled for HIV medication and her life will move on smoothly”. (HIV-infected, female, 25–29 years)*

*“Personally, I would suggest that they visit all pregnant women including other sick people in the community. […] because since they are linked to the hospital and some of us don’t go to the hospital frequently, through them, we get to know any health related information that we need to know”. (HIV-infected, female, 25–29 years)*

Some of the suggested topics to be covered during the visits include reproductive health in general, how to overcome fear related to living with HIV, child health, first aid, tuberculosis, non-communicable diseases and even marital counseling.

*“How to live together using condoms to protect the other partner, I would like to hear more of that. How to take good care of our children and even how to go about getting a child if you don’t have one, and you are HIV-infected”. (HIV-infected, female, 25–29 years)*

*“[…] they will get to solve some marital problems and also keep it confidential, many of us have marital problems even as you see us here. I might have disagreed with my wife, got angry and even threw her drugs in the pit latrine and when am here with you won’t know what happened at home, so when we meet with these mothers then they try to talk to spouses who have marital problems point out mistakes so that such a person changes”. (HIV-infected, Male, 20–24 years)*

Regarding the visits by the cMMs, most women preferred private visits although some pregnant women suggested that their male partners could be involved in at least part of the visit from a cMM.

*“It is good that she will be talking to both of us and therefore we will all read from the same script. We will all listen to what she has to say when she talks to us both”. (HIV-infected, female, 30–34 years)*

There was no universally convenient timing for the visits by the cMMs, since it was assumed that this would have to be individually adjusted based on individual or community preferences. Most individuals suggested that visits should be either in the afternoons or in evenings, when people were finished work for the day and were most likely to be home.

#### Theme 3: ideal characteristics of cMMs

Most of the participants emphasized particularly the need for a cMM to be a person who can protect confidentiality of clients and who is open about her experience living with HIV and her engagement in HIV care and treatment.

*“They should not discuss people’s status. They should maintain confidentiality. She should only tell unrelated stories. The most important characteristic is just confidentiality. She should not say that I went to so, and so’s house and they are all sick”. (HIV-infected, Male, 25–29 years)*

*“The mentor mother, first of all, must be living with HIV. They must be HIV-infected and must be in care. Meaning she has all the information about HIV and more on care so that she can be in a position of telling others the importance of being in care”. (Heath worker, female, 30–34 years)*

Participants also suggested the following desirable characteristics of potential cMMs: middle-aged, knowledgeable, a good educator with good communication skills, friendly, engaging, and familiar with community’s culture and beliefs.

*“Before they start working they need to know the culture of the community. That is one. After knowing the culture of the community she has to know how people live in the community. She has to know what the community members think”. (HIV-infected, Male, 45–49 years)*

#### Theme 4: potential risks of the cMM approach

Potential confidentiality breaches and unwanted disclosure of their client’s HIV-infected status whether due to “slips while gossiping” or inadvertent disclosure, e.g. to their male partners, were frequently identified barriers.

*“What people fear most is being discussed so that is something they should refrain from completely”. (HIV-infected, Male, 30–34 years)*

The participants also felt that cMMs would soon be known in the community as HIV-infected women performing services for the health facility. Hence those visited or associated with cMMs might be assumed to be living with HIV, regardless of their status or intent of visit (personal or clinic business), potentially undermining acceptability and feasibility of the cMM program. This might potentially lead to social isolation and stigmatization of cMMs.

*“…This mentor mother will be questioned or tagged in such a way that if they see a mentor mother entering your compound, then you are an HIV-infected mother. I am sure they will be such in the community and people will be shying away from these mentor mothers.” (Health worker, female, 25–29 years)*

To avoid indirect or accidental HIV-status disclosures, some participants suggested cMMs should perhaps be given small identification documents instead of wearing uniforms or printed T-shirts that might easily identify them as health facility workers.

*“She should come while dressed casually. It is good because when she’ll be moving around no one will know what her agenda is”. (HIV-infected, female, 20–24 years)*

Lack of privacy in the participants’ house was also of concern among most of the participants, male partners included, who felt that they didn’t have private places at their house to meet with cMMs, hence risking disclosure of their status to either the other partner, members of the family or even the entire community.

*“You see when those mentor mothers come to visit you and let’s say someone was passing through home, they may get to hear what you are discussing because you will not be hiding. And those are the people who the moment they leave your home will start spreading the rumors about your HIV status”. (HIV-infected, female, 15–19 years)*

Other participants preferred that these mentor mothers be based solely at the clinic. Those who would prefer visits of a clinic-based mentor mother feared inadvertent or intentional disclosure of their HIV status and stigma.

*“Personally I feel that I should just come to the hospital and finish everything here at the hospital. Because I feel whatever we discuss will spread in the community such that if you go somewhere you hear things about you that you only discussed with the person who visited you at home. You know there are also bad/ill motived people who can just eavesdrop to hear what you are discussing then they go spread what they have heard”. (HIV-infected, female, 15–19 years)*

*“No one will be able to guess that anyone in that group takes ARVs. But when the doctor or nurse visits you at home to teach you, the community will assume that you are on ARVs. Since the nurse too is on ART, people will know that the people she is visiting are on ARVs. Therefore, it is better to meet at the hospital where privacy can be maintained and people can be disguised”. (HIV-infected, Male, 20–24 years)*

When asked if community-based mentor mothers should work in the same community where they live, there were mixed responses due to concerns about inadvertent disclosure.

*“It will be good because they are part of the community and shall be an encouragement to other women since they have been taking their medications appropriately and even their bodies look healthy. They shall be like living testimonies for people to learn from”. (HIV-infected, Male, 30–34 years)*

*“I am a community health worker from this community, and we are supposed to initiate this, they will not agree with me fully, they will say ‘this person is just my neighbor and he is going to tell other people about my status’. So it’s better if mentor mothers are coming from different areas and are stationed in those catchment areas so that there is no any negative view of maybe taking their information out”. (Health worker, male, 35–39 years)*

## Discussion

This study was designed to explore the acceptability of a community-based mentor mother approach in southwestern Kenya. We found that most participants believed that this new intervention, versus a facility-based intervention, would bring the PMTCT services closer to women and concurrently empower the cMMs and as such considered the cMM intervention highly acceptable. We also identified key roles and ideal characteristics of potential cMMs, as well as prominent concerns about inadvertent HIV status disclosure and stigma.

Our study found that the sampled HIV infected women, their male partners as well as the health care providers, viewed the community mentor mother intervention as highly acceptable. This is in agreement with previous studies which have found that task shifting to lay health workers who are also HIV patients is acceptable and results in increased adherence to HIV care [42, 43]. In sub-Saharan Africa, task shifting has been found to be a successful approach for addressing shortages in human resources for HIV services resulting in the ability to offer high-quality, cost-effective care to a larger number of patients than the usual physician-centered model. However, other studies have shown risks involving service delivery from people living with HIV because of stigma [44]. For example a multi-country diagnostic study in Burkina Faso, India and Zambia, found that people leaving with HIV feared that by being involved with AIDS service organizations might ultimately lead to unintended disclosure of their HIV status, resulting in being labeled and discriminated against in society [44–47].

Previous studies have found that the ideal roles of a facility based mentor mother include health education and creating HIV awareness, promotion of HIV counselling and testing of partners and children, ART adherence counselling, tracking patients who do not remain in HIV care, providing counseling on infant feeding, safe sex practices, HIV status disclosure options, adult and infant nutrition, and responding to stigma [47–50]. In addition to these roles, our study participants identified the importance of linking the community and the health facility through the deployment of cMMs.

At the patient level, it is likely that cMMs may provide much needed psychosocial support, as cMMs can assist HIV-infected women in gaining social support and adopting positive coping strategies, both which may support mental health. Indeed, a study in South Africa found that strengthening basic PMTCT services by having support of facility-based mentor mothers could encourage the emotional outlook and hopefulness of HIV-infected pregnant women [48].

From the health facility standpoint, the cMM role involves additional workers who can offer health education, creating HIV awareness, and provide ART adherence counseling. The World Health Organization recognizes the dual issues of overextended health care providers and continuing psychosocial needs of pregnant women living with HIV that are frequently overlooked by the medical system [51]. As such our community-based mentor mothers have the potential to fill a critical gap in the quality and continuum of care for mothers living with HIV.

We found that despite general acceptability of cMMs, certain risks were anticipated, in particular risks of unwanted disclosure of HIV status. Some participants in our study expressed concerns about involuntary HIV-infected status disclosure by cMMs and other confidentiality issues. While this is a genuine concern and in particular in this region that has high HIV-related stigma [6, 36], studies have shown that lay health workers can help facilitate voluntary HIV status disclosure by clients [52, 53]. For example, in South Africa, compared to patients without CHW support, patients with support from CHWs were more likely to disclose their HIV status (58% vs. 42%; *p* = 0.005) [52, 53].

Additionally, mentor mothers have been shown to increase patient satisfaction with services in many settings [54] suggesting that peer supporters might be able to gain trust and provide information not always possible in a more formal provider-patient relationship. They also avoid the barriers of long wait times and short consultation times with each patient in a clinic setting. In our study, as much as participants raised concerns about unwanted disclosure they also expressed belief that the cMMs would be beneficial for stigma reduction. It is clear that concerns regarding unwanted disclosure to community or family members need to be addressed carefully in community mentor mother programs, in order to ensure acceptability and success of these programs.

There is strong evidence of acceptability and impact of lay health workers (including those who are themselves living with HIV) on improving HIV care/treatment outcomes. However, there are a number of challenges in the widespread adoption and scale up. These challenges include lack of formalized recognition within health care systems including lack of remuneration, standardized training, supervision, quality assurance and meaningful participation in decision-making [31, 46, 55–57]. Based on reports from several African settings, mentor mothers have first-hand knowledge of HIV, strong ties to communities, and can gain the trust of other mothers in their communities, making them particularly suited for community-based interventions [19]. The results of a cost-benefit analysis of the m2 m program in Uganda in 2015 showed significant reduction of MTCT, increased retention in care, and increased uptake of infant testing at 18 months for health facilities with m2 m program compared to health facilities without the m2 m program. Additional benefits were observed, including improved psychological well-being among women in care at mentor mother supported health facilities [58].

An important benefit of the proposed intervention in Kenya is that it builds on the already nationally recognized Kenya Mentor Mother Program (KMMP), which is supported by the Kenya National AIDS and STI Control Programme within the primary strategies in Kenya’s national framework for the elimination of mother-to-child transmission of HIV and syphilis (2016–2021) [59]. The KMMP already has standardized hiring, training, and salary standards that are widely accepted [60]. In the long term, we envision a synergistic approach between the facility mentor mothers and the cMMs. The two models can be utilized to ensure continuity of care from the facility (by the health providers and facility mentor mothers) and into the community (by the cMMs). The facility mentor mothers can continue with their roles and duties as defined by the m2 m model at facility level, whereas the cMMs can reinforce health education given at the health facilities, make frequent contact with the mothers in order to ascertain compliance to health education and services received at facility level such as breastfeeding practices, nutrition, adherence to ART and administration of infant prophylaxis. Additionally, the cMMs can link and refer to facilities, mothers, infants and other family members, who would otherwise have waited for their next appointment or not seek healthcare at all.

### Study strengths and limitations

As one of the few studies evaluating the acceptability of a community mentor mother model utilizing women living with HIV based in communities, we explored the potential to provide long-term support for HIV-infected pregnant women with respect to the Option B + approach in Kenya. Perspectives of health care workers, HIV-infected pregnant /postpartum women, and their male partners were explored using qualitative in-depth interviews and focus groups. However, despite the strengths of this approach, this study has several limitations. Our study included women living with HIV only; thus the cMM intervention might become known as a home visiting program for HIV-infected women only and lead to inadvertent disclosure and stigma. In particular, female participants were willing to test for HIV and disclose their HIV status to their partner. These participants could be different from women who were not willing to be tested, those who have not disclosed their status, those who have not utilized antenatal/ postnatal care, or those refusing PMTCT services. Other possible biases that might have occurred include recall bias, given the time frame, and social desirability bias, particularly with regards to HIV stigma. In addition, three counties were represented in this study from southwestern Kenya, thus, the results might not be generalizable to other similar settings.

## Conclusions

Given the current HIV infection rates of children in Kenya, innovative approaches are needed to overcome challenges associated with the scale-up of ART services and Option B+. The cMM intervention combines community-based services with the involvement of women living with HIV both of which have been shown to improve linkage to care, adherence and retention in the PMTCT cascade, and possibly improved maternal and early childhood health outcomes. The findings of this study and early implementation experience suggest that the cMM approach is both acceptable and feasible for supporting HIV service adherence and retention of pregnant/postpartum women in ART care. The effectiveness of the cMM intervention is currently being tested in the MOTIVATE! randomized controlled trial in Kenya.

## Additional files

Additional file 1: **Appendix A** In-depth interview guides pregnant women_English. **Appendix B** In-depth interview guide male partners_English. **Appendix C** In-depth interview participant characteristics form_English. **Appendix D** Focus group discussion guide health care workers/managers_English. **Appendix E** Focus group participant characteristics form_English. (DOC 162 kb)

Additional file 2: **Appendix A** In-depth interview guides pregnant women_Luo. **Appendix B** In-depth interview guide male partners_Luo. **Appendix C** Focus group discussion guide health care workers/managers_Luo. (DOC 168 kb)

## Data Availability

The data generated and analyzed during the current study are available from the corresponding author on reasonable request.

## Abbreviations

ANC: Antenatal care
ART: Antiretroviral treatment
CHW: Community Health Worker
cMM: community-based mentor mother
IDI: In-depth interviews
KMMP: The Kenya Mentor Mother Program (KMMP)
MOTIVATE!: Mother and Infant Visit Adherence and Treatment Engagement
PMTCT: Prevention of mother-to-child transmission of HIV
SSA: Sub-Saharan Africa
