# Supplementary material for: Acceptability of community-based mentor mothers to support HIV-positive pregnant women on antiretroviral treatment in western Kenya: a qualitative study
Source: BMC Pregnancy Childbirth. 2019 Aug 13;19:288. doi: 10.1186/s12884-019-2419-z (PMC6693232; doi:10.1186/s12884-019-2419-z)
Supplement: Supplementary file 1 — Appendix A In-depth interview guides pregnant women_English. Appendix B In-depth interview guide male partners_English. Appendix C In-depth interview participant characteristics form_English. Appendix D Focus group discussion guide health care workers/managers_English. Appendix E Focus group participant characteristics form_English. (DOC 162 kb) [file 12884_2019_2419_MOESM1_ESM.doc]

Supplementary Material 1

Appendix A: In-depth interview guides pregnant women_English

Appendix B: In-depth interview guide male partners_English

Appendix C: In-depth interview participant characteristics form_English

Appendix D: Focus group discussion guide health care workers/managers_English

Appendix E: Focus group participant characteristics form_English

**Appendix A: In-depth interview guides pregnant women_English**

**For women who have tested HIV-positive in ANC**

#### I. INTRODUCTION

My name is ___________________. I am working with the Kenya Medical Research Institute, the University of Colorado, Denver, and the University of Alabama at Birmingham in the United States on a project aiming to improve HIV services in Kenyan communities. We would like to talk to you about your perceptions of prevention of mother-to-child transmission of HIV, HIV treatment for mothers, and some new programs to help pregnant women and their families to live healthy lives. Everything you share during this interview will be kept confidential and will not be shared with your partner afterwards. The information that you provide will be used to inform efforts to strengthen and improve health services in Kenya.

Remember, you don’t have to talk about anything you don’t want to and you may end the interview at any time. This interview will take around one and a half hours. If you have questions you want to ask on other topics, I can assist you to find answers after the interview is over.

(***Go through the informed consent form for in-depth interview participants out loud*** and give the participant a copy. If she agrees to participate, ask her to sign the informed consent form. Complete the participant characteristics form. Ask permission to tape record the discussion, and if she agrees, start the tape recorder AFTER the completion of the participant characteristics form and the introductions part of the discussion. This guide includes the topics to be covered and questions that may be helpful in facilitating the interview. You do NOT have to ask all the questions or follow the order given in the guide.)

**II. DISCUSSION TOPICS**

1. **Perceptions of ART and PMTCT regimens**
   1. Can you tell me what you have heard about treatments for HIV infection? What kinds of treatment are available in your community? Do you believe HIV medications (ART) are effective? Why or why not? Are there other types of treatments that people use for HIV? (Probe for traditional medicines, witchcraft, etc.)
   2. What have you heard about ways to prevent passing the HIV infection from a mother to her infant? Is it possible to prevent? What needs to be done? What else?
   3. Do you believe that taking HIV medication (ART) can prevent mother-to-child transmission of the HIV virus? Why or why not?
   4. How do you think taking ART affects the health of the pregnant woman living with HIV? When should pregnant women start taking ART?
   5. How do you think ART taken by the pregnant mother affects the baby?
2. **Option B+ compared to PMTCT prophylaxis regimens**
   1. What have you heard/observed in terms of how long pregnant women in your community usually take HIV medications in order to prevent passing the HIV infection to the infant? When do they stop taking the medicines?
   2. Have you heard about an option in which the HIV-infected pregnant woman would begin HIV medications (ART) for her own health during pregnancy and continue taking them for life? This option is called Option B Plus. This option lowers the amount of HIV virus in the mother’s body and protects her health, as well as preventing the HIV virus from passing to the infant.
      1. What do you think about that option? What might be the advantages? What about the disadvantages?
      2. Or, would you prefer to only take the medications during pregnancy and breastfeeding and then stop after breastfeeding? Why?
      3. What do you think other pregnant women you know would prefer? What are the reasons?
      4. What do you think husbands/male partners would prefer? Reasons? Other family members?
   3. What would you think about taking ART medications for the rest of your life, even if you are not feeling sick?
   4. Do you think pregnant women in your community would accept Option B+? Why or why not? What about you? What are the reasons for your preference?

**C. Barriers and facilitators to adherence to lifelong ART**

1. What are the challenges to taking HIV medications (ART) every day?
2. If you have taken ART, were there days when you missed taking your HIV medications? Can you tell me about one of those times and what happened? What were the reasons/circumstances that you missed taking your medication? What happened as a result?
3. Do people usually tell their wife/husband/partner that they are taking ART? Why or why not? What about other people in the family? In the wider community?
4. What special challenges do pregnant and postpartum women face in taking their ART medications?
5. What kind of things can help pregnant and postpartum women to take their ART medications every day?

**D. Barriers and facilitators to adherence to retention in HIV care**

1. How important do you think it is to come regularly to the HIV clinic for visits? What are the challenges to coming to the clinic for HIV clinic visits?
2. How often do you come to the HIV clinic (or ANC clinic if integrated services)? What types of services do you receive when you come to the clinic?
3. Please tell me about your participation in any other health services or support groups in the community where you live.
4. Have there been times when you couldn’t make it to your scheduled HIV clinic visit? Can you tell me about one of those times and what happened?
5. Has a health worker ever visited you in your home when you missed an HIV clinic visit? How did you feel about that visit?
6. How do you feel about coming back to the clinic after having missed a visit(s)?
7. What special challenges do pregnant and postpartum women face in coming to the clinic for HIV clinic visits?
8. What kind of things can help pregnant and postpartum women to come to the clinic for their HIV clinic visits?
9. What can health care workers at the clinic do to help you take your medications and return for scheduled visits?

**E. Acceptability of community Mentor Mothers**

We would like to tell you about a program called “community Mentor Mothers”. The mentor mothers would be selected from women in your community who are living with HIV themselves. They will conduct home visits for HIV-positive women and husbands/male partners in their communities in order to assist with safe disclosure, support safe infant feeding, promote safer sex and family planning, encourage early infant testing and follow up, and promote ART adherence and return for HIV care visits.

1. What do you think about this program?
2. Have you ever interacted with a mentor mother at your ANC clinic? Please tell me more about your experience.
3. What do you think about the mentor mothers being based in your community, instead of at the health facility where they are usually based?
4. How do you think people will feel about having these community mentor mothers working in your community?
5. What type of characteristics should these mentor mothers have?
6. What do the mentor mothers need to be careful about when working in the community?

**F. Acceptability of and content of text messages for adherence and retention**

We would like to tell you about a program called “mobile phone text messaging”. In this program mothers receive text messages on their mobile phone to get information about family health and remind them about taking medications and visits to the clinic for themselves and their baby. If desired, the father of the baby can receive messages as well. Women and men can also send a free text message to a health worker, who will call them back to give information and answer any questions.

1. What do you think about this program?
2. Do you have your own mobile phone? If not, do you share a phone with someone?
3. What do you think about receiving messages on family health on your mobile phone? What about messages about HIV medication, HIV clinic visits, and HIV infant testing?
4. What additional topics do you think should be included in the messages?
5. In which language would you be comfortable receiving text messages?
6. Would you be willing to receive these messages on a shared phone? What types of messages would be acceptable to receive on a shared phone?
7. What do you think about fathers/male partners also receiving such messages? Are certain types of messages more appropriate for fathers/male partners?
8. How do you think other women you know might feel about getting such text messages?
9. What do we need to be careful about when designing and sending out these messages? For example, are there topics or words that we should avoid?
10. How often would it be acceptable and helpful to receive these messages? (Daily? Weekly? Monthly?) What time of the day would you prefer to receive messages?
11. Would it be helpful to continue getting these messages after the baby is born? For how long?
12. What kinds of topics would you like to be addressed by messages sent to you after the baby is born?
13. I would like to read you some of the text messages that we are thinking of sending to mothers like yourself after the baby is born. For each one, I will ask you what you understood from the message, how acceptable it would be to receive this message on your mobile phone, how helpful the message might be, and any suggestions you have for improving the message.

- Hi [name]! This week please bring baby [babyname] to clinic for important immunizations to prevent childhood diseases and make sure [babyname] grows up healthy and strong. You will also be counseled on how to keep your baby [boy/girl] healthy.
- Hi [name]! Congratulations for visiting the clinic this week. Please call or flash XXXXXXXXX if you have questions about your health or the health of your baby. We are here to help you.
- Hi [name]! Remember the health of the mother is very important for the health of the baby and the whole family. Please come to the clinic for regular check-ups for yourself and please call or flash XXXXXXXXXX if you have any questions about your health.

**III. CLOSING**

Thank you very much for your time. Your responses will be very helpful for improving the health of Kenyan families.

(Correct any important misconceptions and provide referrals to PMTCT or ARV services, if appropriate.)

## Appendix B: In-depth interview guide male partners_English

**For husbands/male partners of women who have tested HIV-positive in ANC**

#### I. INTRODUCTION

My name is ___________________. I am working with the Kenya Medical Research Institute, the University of Colorado, Denver, and the University of Alabama at Birmingham in the United States on a project aiming to improve HIV services in Kenyan communities. We would like to talk to you about your perceptions of prevention of mother-to-child transmission of HIV, HIV treatment for mothers, and some new programs to help pregnant women and their families to live healthy lives. Everything you share during this interview will be kept confidential and will not be shared with your partner afterwards. The information that you provide will be used to inform efforts to strengthen and improve health services in Kenya.

Remember, you don’t have to talk about anything you don’t want to and you may end the interview at any time. This interview will take around one and a half hours. If you have questions you want to ask on other topics, I can assist you to find answers after the interview is over.

(***Go through the informed consent form for in-depth interview participants out loud*** and give the participant a copy. If he agrees to participate, ask him to sign the informed consent form. Complete the participant characteristics form. Ask permission to tape record the discussion, and if he agrees, start the tape recorder AFTER the completion of the participant characteristics form and the introductions part of the discussion. This guide includes the topics to be covered and questions that may be helpful in facilitating the interview. You do NOT have to ask all the questions or follow the order given in the guide.)

**II. DISCUSSION TOPICS**

1. **Perceptions of ART and PMTCT regimens**
   - - 1. Can you tell me what you have heard about treatments for HIV infection? What kinds of treatment are available in your community? Do you believe HIV medications (ART) are effective? Why or why not? Are there other types of treatments that people use for HIV? (Probe for traditional medicines, witchcraft, etc.)
       2. How does taking ART affect people who take these medicines? Positive effects? Negative effects?
       3. How do people in your community view persons who are on ART?
       4. What have you heard about ways to prevent passing the HIV infection from a mother to her infant? Is it possible to prevent? What needs to be done? What else?
   1. Do you believe that taking HIV medication (ART) can prevent mother-to-child transmission of the HIV virus? Why or why not?
   2. How do you think taking ART affects the health of the pregnant woman living with HIV? When should pregnant women start taking ART?
   3. How do you think ART taken by the pregnant mother affects the baby?
2. **Option B+ compared to PMTCT prophylaxis regimens**
   - - 1. What have you heard/observed in terms of how long pregnant women in your community usually take HIV medications in order to prevent passing the HIV infection to the infant? When do they stop taking the medicines?
       2. Have you heard about an option in which the HIV-infected pregnant woman would begin HIV medications (ART) for her own health during pregnancy and continue taking them for life? This option is called Option B Plus. This option lowers the amount of HIV virus in the mother’s body and protects her health, as well as preventing the HIV virus from passing to the infant.
     1. What do you think about that option? What might be the advantages? What about the disadvantages?
     2. Or, would you prefer that your wife/female partner only take the medications during pregnancy and breastfeeding and then stop after breastfeeding? Why?
     3. What do you think your wife/female partner would prefer? What are the reasons?
3. What would you think about your wife/female partner taking ART medications for the rest of her life, even if she is not feeling sick?
4. Do you think pregnant women in your community would accept Option B+? Why or why not? What about you? What are the reasons for your preference?

**C. Barriers and facilitators to adherence to lifelong ART**

1. What are the challenges to taking HIV medications (ART) every day?
2. Do people usually tell their wife/husband/partner that they are taking ART? Why or why not? What about other people in the family? In the wider community?
3. What special challenges do men face in taking their ART medications?
4. What special challenges do pregnant and postpartum women face in taking their ART medications?
5. What kind of things do you think could help people to take their ART medications every day?

If the man has/is taking ART:

1. What were/are the challenges of taking ART for you?
2. Were there days when you missed taking your HIV medications? Can you tell me about one of those times and what happened? What were the reasons/circumstances that you missed taking your medication? What happened as a result?

**D. Barriers and facilitators to adherence to retention in HIV care**

1. How important do you think it is to come regularly to the HIV clinic for visits? What are the challenges people face in coming to the clinic for HIV clinic visits?
2. What special challenges do men face in coming to the clinic for HIV clinic visits?
3. What special challenges do pregnant and postpartum women face in coming to the clinic for HIV clinic visits?
4. What kind of things can help people to come to the clinic for their HIV clinic visits?

If the man is an HIV clinic patient:

1. What are the challenges for you in coming to the clinic for HIV clinic visits?
2. How often do you come to the HIV clinic? What types of services do you receive when you come to the clinic?
3. Please tell me about your participation in any other health services or support groups in the community where you live.
4. Have there been times when you couldn’t make it to your scheduled HIV clinic visit? Can you tell me about one of those times and what happened?
5. Has a health worker ever visited you in your home when you missed an HIV clinic visit? How did you feel about that visit?
6. How do you feel about coming back to the clinic after having missed a visit(s)?
7. What can health care workers at the clinic do to help you take your medications and return for scheduled visits?

**E. Acceptability of community Mentor Mothers**

We would like to tell you about a program called “community Mentor Mothers”. The mentor mothers would be selected from women in your community who are living with HIV themselves. They will conduct home visits for HIV-positive women and husbands/male partners in their communities in order to assist with safe disclosure, support safe infant feeding, promote safer sex and family planning, encourage early infant testing and follow up, and promote ART adherence and return for HIV care visits.

1. What do you think about this program?
2. Have you ever interacted with a mentor mother at your ANC clinic? Please tell me more about your experience.
3. What do you think about the mentor mothers being based in your community, instead of at the health facility where they are usually based?
4. How do you think people will feel about having these community mentor mothers working in your community?
5. What type of characteristics should these mentor mothers have?
6. What do the mentor mothers need to be careful about when working in the community?

**F. Acceptability of and content of text messages for adherence and retention**

We would like to tell you about a program called “mobile phone text messaging”. In this program mothers receive text messages on their mobile phone to get information about family health and remind them about taking medications and visits to the clinic for themselves and their baby. If desired, the father of the baby can receive messages as well. Women and men can also send a free text message to a health worker, who will call them back to give information and answer any questions.

1. What do you think about this program?
2. Do you have your own mobile phone? If not, do you share a phone with someone? Does your wife/female partner have her own mobile phone?
3. What do you think about receiving messages on family health on your mobile phone? What about messages about HIV medication, HIV clinic visits, and HIV infant testing?
4. What additional topics do you think should be included in the messages?
5. Would you be willing to receive these messages on a shared phone? What types of messages would be acceptable to receive on a shared phone?
6. What do you think about fathers/male partners also receiving such messages? Are certain types of messages more appropriate for fathers/male partners?
7. How do you think other men you know might feel about getting such text messages?
8. What do we need to be careful about when designing and sending out these messages? For example, are there topics or words that we should avoid?

How often would it be acceptable and helpful to receive these messages? (Daily? Weekly? Monthly?) What time of the day would you prefer to receive messages?

1. Would it be helpful to continue getting these messages after the baby is born? For how long?
2. What kinds of topics would you like to be addressed by messages sent to you after the baby is born?
3. I would like to read you some of the text messages that we are thinking of sending to fathers like yourself after the baby is born. For each one, I will ask you what you understood from the message, how acceptable it would be to receive this message on your mobile phone, how helpful the message might be, and any suggestions you have for improving the message.

- Hi [name]! This week please bring baby [babyname] to clinic for important immunizations to prevent childhood diseases and make sure [babyname] grows up healthy and strong. You will also be counseled on how to keep your baby [boy/girl] healthy.
- Hi [name]! Congratulations for visiting the clinic this week. Please call or flash XXXXXXXXX if you have questions about your health or the health of your family. We are here to help you.
- Hi [name]! Remember the health of the father is very important for the health of the baby and the whole family. Please come to the clinic for regular check-ups for yourself and please call or flash XXXXXXXXXX if you have any questions about your health

**III. CLOSING**

Thank you very much for your time. Your responses will be very helpful for improving the health of Kenyan families.

(Correct any important misconceptions and provide referrals to PMTCT or ARV services, if appropriate.)

## Appendix C: In-depth interview participant characteristics form_English

**OPTION B+ STUDY QUALITATIVE IN-DEPTH INTERVIEWS PARTICIPANT CHARACTERISTICS FORM**

**PARTICIPANT CHARACTERISTICS FORM FOR IN-DEPTH INTERVIEWS**

| Study ID# |  |
| --- | --- |
| Type of participant  (Circle one) | 1. Pregnant woman 2. Male partner of pregnant woman |
| Name of community / health facility where recruited |  |
| Age |  |
| Birth place |  |
| Educational level  (Circle one) | 1. Less than primary 2. Completed primary 3. Some secondary 4. Completed secondary 5. More than secondary |
| Marital status  (Circle one) | 1. Single, never married 2. Married 3. Widowed/divorced/separated |
| Currently living with a spouse/partner? (Y/N) |  |
| Type of relationship  (Circle one) | 1. Monogamous 2. Polygamous |
| Type of partnership with spouse/partner  (Circle one) | 1. Concordant 2. Discordant |
| Number of living children |  |
| Main occupation |  |
| If pregnant, weeks of pregnancy |  |
| Was this pregnancy planned? (Y/N) |  |
| Other notes |  |

## Appendix D: Focus group discussion guide health care workers/ managers_English

**For service providers (health professionals, lay health workers, community mobilizers, and community leaders)**

#### INTRODUCTION

My name is ___________________. We am working with the Kenya Medical Research Institute, the University of Colorado, Denver, and the University of Alabama at Birmingham in the United States on a project aiming to improve HIV services in Kenyan communities. We would like to talk to you about your perceptions of options for prevention of mother-to-child transmission of HIV, HIV treatment for mothers, and some new programs to help pregnant women and their families to live healthy lives. Everything you share during this focus group will be kept confidential. The information that you provide will be used to inform efforts to strengthen and improve health services in Kenya.

Remember, you don’t have to talk about anything you don’t want to and you may end the interview at any time. This discussion will take around one and a half hours. If you have questions you want to ask on other topics, I can assist you to find answers after the focus group discussion is over.

(***Go through the information sheet for focus group discussions out loud*** and give each participant a copy. Ask permission to tape record the discussion, and if they agree, start the tape recorder AFTER the introductions part of the discussion. This guide includes the topics to be covered and questions that may be helpful in facilitating the focus group discussion. You do NOT have to ask all the questions or follow the order given in the guide. Major topic areas and questions are indicated.

#### II. INTRODUCTIONS

First let’s get acquainted. Let’s go around the circle and each person can introduce himself. You can tell us your first name (or name you would like to use in this group discussion), what type of service provider you are, and anything else about yourself that you would like to tell the group. (Members of the research team should also introduce themselves. If the group agreed to the tape recording, you may start recording after this section of the discussion.)

**III. DISCUSSION TOPICS**

1. **Community Perceptions of ART and PMTCT regimens**
   1. How do people in this community view treatments for HIV infection (ART)? Do they believe those treatments are effective? Why or why not? Are there other types of treatments that people use for HIV? (Probe for traditional medicines, witchcraft, etc.)
   2. What do people in the community believe about the positive effects of ART? What do they believe about the negative effects?
   3. How do people in this community view persons who are on ART?
   4. How do people feel about pregnant women taking ART to prevent mother-to-child transmission?
2. **Option B+ compared to PMTCT prophylaxis regimens**
   1. Have you heard about an option in which the HIV-infected pregnant woman would begin HIV medications (ART) for her own health during pregnancy and continue taking them for life, regardless of her CD4 count or state of HIV disease? This option is called Option B Plus. This option lowers the amount of HIV virus in the mother’s body and protects her health, as well as preventing the HIV virus from passing to the infant.
      1. What do you think about that option? What might be the advantages? What about the disadvantages?
      2. Or, do you think women in this community would prefer to only take the medications during pregnancy and breastfeeding and then stop after breastfeeding? Why?
      3. What do you think husbands/male partners would prefer? Reasons? Other family members?
   2. How do you think women in this community would feel about taking ART medications for the rest of their lives, even if they are not feeling sick?
   3. Do you think pregnant women in your community would accept Option B+? Why or why not? How could it be promoted in the community?
   4. What do you think is the best option for HIV-positive pregnant women? What are the reasons for your preference? Do you have any concerns about Option B+?
   5. What might the challenges of Option B+ be from the perspective of service provision? What are the challenges to providing this option at your health facility? Supplies of drugs? Loss-to-follow-up? Long-term monitoring of clients’ health? Other?

**C. Barriers and facilitators to adherence to lifelong ART**

1. What are the challenges that people in this community face in taking HIV medications (ART) every day?
2. Do people usually tell their wife/husband/partner that they are taking ART? Why or why not? What about other people in the family? In the wider community?
3. What special challenges do pregnant and postpartum women face in taking their ART medications?
4. What kind of things can help pregnant and postpartum women to take their ART medications every day?

**D. Barriers and facilitators to adherence to retention in HIV care**

1. Do your HIV-positive clients think that it is important to come for regular HIV clinic visits? What are the challenges that people in this community face in coming to the clinic for HIV clinic visits?
2. Please tell me about any other health services or support groups that are available in the community here (outside of the health facility).
3. Do health workers visit people in their homes when they miss an HIV clinic visit? How do those visits work? What are the challenges? How do you think clients feel about coming back to the clinic after they have missed a visit(s)?
4. What special challenges do pregnant and postpartum women face in coming to the clinic for HIV clinic visits?
5. What kind of things can help pregnant and postpartum women to come to the clinic for their HIV clinic visits?
6. What can health care workers at the clinic do to help clients take their medications and return for scheduled visits?

**E. Acceptability of community Mentor Mothers**

We would like to tell you about a program called “community Mentor Mothers”. The mentor mothers would be selected from women in the community who are living with HIV themselves and would be based in the community (not at the health facility). They will conduct home visits for HIV-positive women and husbands/male partners in their communities in order to assist with safe disclosure, support safe infant feeding, promote safer sex and family planning, encourage early infant testing and follow up, and promote ART adherence and return for HIV care visits.

1. What do you think about this program?
2. Do you have mentor mothers based at your ANC clinic? Please tell me about your experiences with mentor mothers.
3. What do you think about the mentor mothers being based in the community, instead of at the health facility where they are usually based?
4. How do you think people will feel about having these community mentor mothers working in your community?
5. What type of characteristics should these mentor mothers have?
6. What do the mentor mothers need to be careful about when working in the community?

**F. Acceptability of and content of text messages for adherence and retention**

We would like to tell you about a program called “mobile phone text messaging”. In this program mothers receive text messages on their mobile phone to get information about family health and remind them about taking medications and visits to the clinic for themselves and their baby. If desired, the father of the baby can receive messages as well. Women and men can also send a free text message to a health worker, who will call them back to give information and answer any questions.

1. What do you think about this program?
2. Do women in this community usually have their own mobile phones? If not, do they share a phone with someone? What proportion would you say share phones?
3. Do you think it would be acceptable for women to receive messages on family health on their mobile phones? What about messages about HIV medication, HIV clinic visits, and HIV infant testing?
4. What additional topics do you think should be included in the messages?
5. Would they be willing to receive these messages on a shared phone? What types of messages would be acceptable to receive on a shared phone?
6. What do you think about fathers/male partners also receiving such messages? Are certain types of messages more appropriate for fathers/male partners?

What do we need to be careful about when designing and sending out these messages? For example, are there topics or words that we should avoid?

1. How often would it be acceptable and helpful for women to receive these messages? (Daily? Weekly? Monthly?) What time of the day do you think men/women would prefer for receiving messages?
2. Would it be helpful for mothers and fathers to continue getting these messages after the baby is born? For how long?
3. What kinds of topics would you like to be addressed by messages sent after the baby is born?
4. I would like to read you some of the text messages that we are thinking of sending to mothers and fathers after the baby is born. For each one, I will ask you what you understood from the message, how acceptable you think it would be to receive this message on a mobile phone, how helpful the message might be, and any suggestions you have for improving the message.

- For both mothers and fathers: Hi [name]! This week please bring baby [babyname] to clinic for important immunizations to prevent childhood diseases and make sure [babyname] grows up healthy and strong. You will also be counseled on how to keep your baby [boy/girl] healthy.
- For both mothers and fathers: Hi [name]! Congratulations for visiting the clinic this week. Please call or flash XXXXXXXXX if you have questions about your health or the health of your family. We are here to help you.
- For mothers: Hi [name]! Remember the health of the mother is very important for the health of the baby and the whole family. Please come to the clinic for regular check-ups for yourself and please call or flash XXXXXXXXXX if you have any questions about your health.
- For fathers: Hi [name]! Remember the health of the father is very important for the health of the baby and the whole family. Please come to the clinic for regular check-ups for yourself and please call or flash XXXXXXXXXX if you have any questions about your health.

1. What other types of messages would you suggest we send to help women on Option B+ (and their infants) with adhering to their medications and coming to regular HIV care visits at the clinic?

**G. Other suggestions**

1. What other suggestions do you have to ensure the success of Option B+ in this community/health facility?

**IV. CLOSING**

Thank you very much for your time. Your responses will be very helpful for improving the health of Kenyan families.

## APPENDIX E: Focus group participant characteristics form

**OPTION B+ STUDY FOCUS GROUP PARTICIPANT CHARACTERISTICS FORM**

PLACE:

MODERATOR:

NOTE TAKER:

DATE:

Beginning time: Ending time:

TYPE OF GROUP:

CHARACTERISTICS OF PARTICIPANTS

|  | 1 | 2 | 3 | 4 | 5 | 6 | 7 | 8 |
| --- | --- | --- | --- | --- | --- | --- | --- | --- |
| Age |  |  |  |  |  |  |  |  |
| Gender |  |  |  |  |  |  |  |  |
| Educational Level |  |  |  |  |  |  |  |  |
| Marital status |  |  |  |  |  |  |  |  |
| Religion |  |  |  |  |  |  |  |  |
| Current job / role in community |  |  |  |  |  |  |  |  |
| Length of time in current job/role |  |  |  |  |  |  |  |  |
| Number of living children |  |  |  |  |  |  |  |  |
