# Supplementary material for: Acceptability of community-based mentor mothers to support HIV-positive pregnant women on antiretroviral treatment in western Kenya: a qualitative study
Source: BMC Pregnancy Childbirth. 2019 Aug 13;19:288. doi: 10.1186/s12884-019-2419-z (PMC6693232; doi:10.1186/s12884-019-2419-z)
Supplement: Supplementary file 2 — Appendix A In-depth interview guides pregnant women_Luo. Appendix B In-depth interview guide male partners_Luo. Appendix C Focus group discussion guide health care workers/managers_Luo. (DOC 168 kb) [file 12884_2019_2419_MOESM2_ESM.doc]

Supplementary Material 2

Appendix A: In-depth interview guides pregnant women_Luo

Appendix B: In-depth interview guide male partners_Luo

Appendix C: Focus group discussion guide health care workers/managers_Luo

### Appendix A: In-depth interview guides pregnant women_Luo

**For women who have tested HIV-positive in ANC**

#### I. INTRODUCTION

Nyinga en ___________________. Atiyo gi Kar Nonro mar Thieth ma Kenya (KEMRI), Mbalariany ma Kolorado, Denver, kod Mbalariany ma Alabama, Birmingham man e piny Amerka e chenro ma dwaro keto maber kony mag thieth mag kute mag ayaki e oganda man e Kenya. Dwaher mar wuoyo kodi kuom pachi kuom geng’o landruok mag kute mag ayaki ne nyithindo ka wuok kuom mine gi, thieth mar kute mag ayaki ne jomamine, kod chenro moko manyien ma konyo mine mapek kod familia gi mondo odag ngima maber maonge tuoche . Gimoro amora ma iwacho e kinde twak ni ok bi nyis jahera ni bang’e. Weche ma ichiwo ibiro ti godo e tego kendo keto maber chenro mag thieth e Kenya.

Onego ing’e ni, Ok ochuno ni nyaka iwuo kuom gima ok idwar wuoyo e kendo inyalo weyo twak ni e saa asaya. Twak ni biro kawo madirom saa achiel gi nus. Ka in gi penjo ma diher penjo kuom weche mamoko, anyalo konyo yudo dwoko bang’ ka twak oserumo.

(***Go through the informed consent form for in-depth interview participants out loud*** and give the participant a copy. If she agrees to participate, ask her to sign the informed consent form. Complete the participant characteristics form. Ask permission to tape record the discussion, and if she agrees, start the tape recorder AFTER the completion of the participant characteristics form and the introductions part of the discussion. This guide includes the topics to be covered and questions that may be helpful in facilitating the interview. You do NOT have to ask all the questions or follow the order given in the guide.)

**II. DISCUSSION TOPICS/thor weche twak**

1. **Pachi kuom yedhe ma itiyo go e gayo kute mag ayaki kod ma geng’o landruok kute mag ayaki ka owuok kuom miyo ka dhi ne nyathine**
   1. Bende inyalo nyisa gik ma isewinjo kuom thieth mag kute mag ayaki? Gin kit thieth mage ma yudore e oganda u? Bende in gi geno ni yedhe ma gayo kute mag ayaki(ART) tiyo? Nang’o kata nang’o ok in gi geno? Bende nitie kit thieth mamoko ma ji tiyo godo kuom kute mag ayaki? (Probe for traditional medicines, witchcraft, etc.)
   2. Gin ang’o ma isewinjo kuom yore mag geng’o lando kute mag ayaki ka oa kuom miyo ka dhi kuom nyathine? Bende inyalo geng’e? Ang’o ma onego otim? Ang’o kendo?
   3. Bende in gi geno ni mwonyo yedhe ma gayo kute mag ayaki (ART) nyalo geng’o lando kute mag ayaki ka owuok kuom miyo ka dhi kuom nyathine ? Nang’o kata nang’o ok in gi geno?
   4. Iparo ni mwonyo yedhe ma gayo kute mag ayaki (ART) timo nade ngima mar miyo mapek man kod kute mag ayaki? En thulo mane ma mine mapek onego ochak mwonyo yedhe ma gayo kute mag ayaki?
   5. Iparo ni yedhe ma gayo kute mag ayaki (ART) ma imwonyo gi mine mapek man gi kute mag ayaki timo nyathi nadi?
2. **Pimo thieth mar Option B+ kod thieth mar geng’o landruok kute mag ayaki ka wuok kuom miyo ka dhi ne nyathine**
   1. Gin ang’o ma isewinjo/neno kalure gi thuolo ma mine man e oganda u tiyo kod yedhe mag kute mag ayaki mondo gigeng’ lando kute mag ayaki ne nyithindgi mayom ? Giweyo mwonyo yedhe gi e thuolo mane?
   2. Bende isewinjo wach kuom yiero moro ma mine man kod kute mag ayaki chako yedhe ma gayo kute mag ayaki (ART) ne ngimane owuon e kinde ma en gi ich kendo dhi nyime kod mwonyo gi e ngimane duto? Yiero ni iluongo ni Option B Plus. Yiero no dwoko chien kwan mag kute mag ayaki e dend miyo kendo rito ngimane, ka bende ogeng’o kute mag ayaki kuom landore ne nyathi mayom.
      1. Iparo nang’o kuom yiero no? Gin ber mage ma onyalo bedo go? Gin rach mage ma onyalo bedo go?
      2. Kata, diher mondo imwony yedhe e kinde ma in gi ich kod ka idhodho kendo iweyo bang’ ka isedhodho nyathi? Nang’o?
      3. Iparo ni jomamine mamoko ma ing’eyo nyalo hero ang’o? Nikech ang’o?
      4. Iparo ang’o joute/johera ma chuo nyalo hero ? Nang’o? Jo anyuola kata familia u mamoko?
   3. Iparo ang’o kuom mwonyo yedhe ma gayo kute mag ayaki (ART) kuom ngimani duto, kata ka ok iwinjo ka ituo?
   4. Bende iparo ni mine mapek e oganda u nyalo rwako Option B+? Nang’o kata nang’o ok ginyalo rwake? Gin ang’o ma omiyo in gi yiero no?

**C. Gik ma mono kod ma konyo mwonyo yedhe ma gayo kute mag ayaki kaka dwarore e ngima ng’ato duto**

1. Gin pek mage man e mwonyo yedhe mag gayo kute mag ayaki (ART) pile pile?
2. Ka isemwonyo yedhe ma gayo kute mag ayaki (ART), bende nitie odiochienge moko ma iweyo ma ok imwonyo yedhe gi mag kute mag ayaki? Bende inyalo nyisa kuom achiel kuom odiochienge go kod gima ne otimore? Gin gik mage/ang’o mane omiyo ilewo mwonyo yedhe gi ? Ang’o mane otimore nikech mano?
3. Bende ji nyiso ga monde gi/chuo gi/johera gi ni gimwonyo yedhe ma gayo kute mag ayaki (ART) ? Nang’o kata nang’o ok ginyis gi? To ji mamoko e familia/anyuola? E oganda?
4. Gin pek mage makende ma jomamine mapek kod mosenyuol bedo godo kuom mwonyo yedhe gi ma gayo kute mag ayaki (ART)?
5. Gin gik mage ma nyalo konyo jomamine mapek kod jomamine ma osenyuol mondo omwony yedhe gi ma gayo kute mag ayaki pile ka pile?

**D. Gik ma mono kod ma konyo e bedo kaka dwarore e rit mar kute mag ayaki**

1. Iparo ni en gimaduong moromo nadi mondo ji odhi kaka dwarore e klinik mar kute mag ayaki ne limbe ? Gin pek mage ma ji bedo godo e dhi e limbe mag klinik mar kute mag ayaki?
2. Ibiro ga moluwore ma nadi e klinik mar kute mag ayaki (kata klinik mar jomamine gi nyithindo ka po ni kony go oket kanyakla)? Gin kit kony mage ma iyudo ka ibiro e klinik?
3. Yie inyisa kuom bedo mari e kony mamoko mag thieth kata grube mag siro ji e oganda ma idak e.
4. Bende isebedo gi thuolo moko ma ne ok inyal chopo ne limbe gi mochan mag klinik mar kute mag ayaki? Nyisa kuom achiel kuom thuolo kod gima ne otimore?
5. Bende jatij thieth osebiro limi e dalani ka ilewo ne limbe mar klinik mar kute mag ayaki? Ne iwinjo nadi kuom limbe no?
6. Iwinjo nadi kuom duogo e klinik bang’ ka iselewo ne limbe ?
7. Gin pek mage makende ma ma jomamine mapek kod mosenyuol yudo e biro klinik ne limbe mag klinik mar ayaki Gin gik mage ma nyalo konyo mine mapek kod mosenyuol mondo obi e klinik ne limbe mag klinik mar kute mag ayaki?
8. Gin ang’o ma jotij thieth e klinik nyalo timo mondo okonyi mwonyo yedhe gi kendo iduog ne limbe ma ochan?

**E. Rwako mine man e oganda ma tiego jomamoko**

Dwaher mar nyisi kuom chenro ma iluongo ni “community Mentor Mothers”. Mine ma tiego ji ibiro yier kuom jomamine man e oganda u kendo ma odak kod kute mag ayaki. Gibiro timo limbe ma dala ne jomamine man kod kute mag ayaki gi chuo gi/johera gi machuo e oganda gi mondo gikony e yangruok e yo maber, siro yor pidho nyathi ma ok kete e thuolo mar gamo kute, medo siro bedo e achiel gi rageng kod komo nyuol, jiwo ji mondo opim nythindgi mayom chon kendo timo ne ji limbe, kod jiwo mwonyo yedhe ma gayo kute mag ayaki kaka dwarore kod dwogo e limbe mag rit mar kute mag ayaki .

1. Iparo nadi kuom chenro ni?
2. Bende isegatudori kod miyo ma tiego ji e klinik mari mar jomamine gi nyithindo? Yie inyisa mthoth kuom kaka ne obedo.
3. Iparo nadi kuom mine ma tiego ji go bedo e oganda, kar bedo e kar thieth ma gibedo ga e?
4. Iparo ni ji biro neno nadi kuom bedo gi mine ma tiego ji e gweng’ go e oganda u?
5. Gin kido mage ma jomamine ma tiego ji e gweng’ gi onego obed godo?
6. Gin ang’o ma mine ma tiego jomamoko gi onego ong’I ahinya ka gitiyo e oganda

**F. Rwako kod wach ma onego obed e ote machwok mar sime motudore kod mwonyo yath kaka dwarore kendo keto ji dhi e limbe kaka dwarore**

Dwaher mar nyisi kuom chenro ma iluongo ni “mobile phone text messaging”. E chenro ni mine biro yudo ote machwok mar sime e simbe gi mag ong’we yamo mondo giyud weche kuom ngima familia kendo paro ne gi ni onego gimwony yath kod limbe mag klinik ne gin kod nyithindgi. Ka gidwaro, wuon nyathi bende nyalo yudo ote machwok no bende. Jomamine gi jomachuo bende nyalo oro ote machwok maonge chudo ne jatij thieth, mar biro goyo ne gi sime mondo onyis gi gik ma gidwaro ng’eyo kendo dwoko penjo moro amora.

.

1. Iparo nade kuom chenro ni?
2. Bende in gi simb ong’we yamo ma mari? Ka ionge go, bende iriwo sime gi ng’ato machielo?
3. Iparo nade kuom yudo ote machwok kuom ngima familia e simbi mar ong’we yamo (mobail)? To kuom ote machwok kuom yedhe mag kute mag ayaki, limbe mag klinik mar kute mag ayaki, kod pimo nyithindo mayom ne kute mag ayaki?
4. Bende inyalo dwaro mondo iyud ote machwok gi e sime ma uriwo? Gin kit ote machwok mage ma nyalo bedo maber yudo e sime ma uriwo?
5. Iparo nadi kuom wuone/johera machuo bende yudo ote machwok machal kamago ? Bende kit ote moko machwok nyalo bedo ni ber ne wuone/johera machuo ?
6. Iparo ni jomamine mamoko ma ing’eyo nyalo winjo nadi kuom yudo ote machwok macahl kamago?
7. Gin gik mage ma onego wang’Iwatang’ne ahinya ka wandiko kendo oro ote machwok gi?kuom ranyisi, bende nitiere thor weche moko kata weche moko ma onego ik tigo?
8. Iparo ni ote machwok mag sime gi onego obi maluwore marom nadi kendo ginyalo konyo ka gibiro moluwore machal nadi? (Pile pile? Dichiel e juma? Dichiel e dwe?) En seche mage e odio chien’g madiher mar yudo ote machwok?
9. Bende ginyalo konyo yudo ote machwok gi bang’ ka nyathi osenyuol? Kuom thuolo maromo nadi?
10. Gin thor weche mage monego ote machuok mioroni twagie bang ka isenyuol/ka nyathi osenyuol?
11. **Daher mar somoni moko kuom ote machwok ma waparo mar oro ne mine kaka in bang ka nyathi osenyuol. Kuom moro ka moro abiro penji gima iwinjo/fuono e ote machwok no,bende ibiro yie mar yudo ote machwok gi e simbi mar ongwe yamo, bende ote machwok gi biro bedo gi kony, kod paro moro amora ma dibedgo e keto makare ote machwok gi.**

- Amosi (name) wigni akwayi ni ikel nyathi ma(name) e klinik e chanjo mondo ogeng’ touché mayudo nyithindo kapod kithindo kendo ma biro miyo nyathi ma(name) dong makare kendo ma otegno, bende ibiro bedo gi twak mar hocho kuom rito nyathi ma(nyako/wuoyi) e yo makare
- Amosi (name) Itimo maber kuom limo klinik wigni. Asayi ni gochi kata e flash XXXXXXXX ka in kod penjo kuom ngima ni kata gima mar nyathini. Wantie ka mar konyi.
- Amosi (name) bed ka ingeyo ni gima mar miyo en gima ogen kod gima mar nyathi kaachiel gi familia duto. Asayi ni mondo ibi e klinik ne neno ma kinde ka kinde ne in iwuon kendo igochi kata iflash XXXXXXXX ka in kod penjo moro amora kuom gima ni.

**III. CLOSING**

Ero kamano kuom thuolo ni. Dwoko gi biro konyo ahinya kuom keto ngima familia ma Kenya maber .

(Correct any important misconceptions and provide referrals to PMTCT or ARV services, if appropriate.)

### Appendix B: In-depth interview guide male partners_Luo

**For husbands/male partners of women who have tested HIV-positive in ANC**

#### I. INTRODUCTION

Nyinga en ___________________. Atiyo gi Kar Nonro mar Thieth ma Kenya (KEMRI), Mbalariany ma Kolorado, Denver, kod Mbalariany ma Alabama, Birmingham man e piny Amerka e chenro ma dwaro keto maber kony mag thieth mag kute mag ayaki e oganda man e Kenya. Dwaher mar wuoyo kodi kuom pachi kuom geng’o landruok mag kute mag ayaki ne nyithindo ka wuok kuom mine gi, thieth mar kute mag ayaki ne jomamine, kod chenro moko manyien ma konyo mine mapek kod familia gi mondo odag ngima maber maonge tuoche . Gimoro amora ma iwacho e kinde twak ni ok bi nyis jahera ni bang’e. Weche ma ichiwo ibiro ti godo e tego kendo keto maber chenro mag thieth e Kenya.

Onego ing’e ni, Ok ochuno ni nyaka iwuo kuom gima ok idwar wuoyo e kendo inyalo weyo twak ni e saa asaya. Twak ni biro kawo madirom saa achiel gi nus. Ka in gi penjo ma diher penjo kuom weche mamoko, anyalo konyo yudo dwoko bang’ ka twak oserumo.

(***Go through the informed consent form for in-depth interview participants out loud*** and give the participant a copy. If he agrees to participate, ask him to sign the informed consent form. Complete the participant characteristics form. Ask permission to tape record the discussion, and if he agrees, start the tape recorder AFTER the completion of the participant characteristics form and the introductions part of the discussion. This guide includes the topics to be covered and questions that may be helpful in facilitating the interview. You do NOT have to ask all the questions or follow the order given in the guide.)

**II. DISCUSSION TOPICS**

1. **Pachi kuom yedhe ma itiyo go e gayo kute mag ayaki kod ma geng’o landruok kute mag ayaki ka owuok kuom miyo ka dhi ne nyathine**
   1. Nyisa gik ma isewinjo kuom thieth ne kute mag ayaki? Gin kit thieth mage ma yudore e oganda u? Bende in gi geno ni yedhe ma gayo kute mag ayaki tiyo? Nang’o kata Nang’o ok ipar kamano? Bende nitie kit thieth mamoko ma jit tiyo go e thiedho kute mag ayaki? (Probe for traditional medicines, witchcraft, etc.)
   2. Ere kaka mwonyo yedhe ma gayo kute mag ayaki timo jogo ma tiyo kod gi? Gik ma gitimo mabeyo? Gik ma gitimo maricho?
   3. Ere kaka ji e oganda u neno jogo man e thieth mar kute mag ayaki?
   4. Gin gik mage ma isewinjo kuom yore ma igeng’o godo landruok kute mag ayaki kowuok kuom miyo ka dhi ne nyathine? Bende inyalo geng’e? Ang’o ma dwarore ni mondo otim? Ang’o kendo?
   5. Bende in gi geno ni tiyo kod yedhe ma gayo kute mag ayaki (ART) nyalo geng’o landruok mag kute mag ayaki kowuok kuom miyo ne nyathine ? Nang’o kata nang’o ok in gi geno?
   6. Iparo ni tiyo kod yedhe ma gayo kute mag ayaki timo ngima miyo mapek man kod kute mag ayaki nadi? En thuolo mane ma mine mapek onego ochak mwonyo yedhe ma gayo kute mag ayaki?
   7. Iparo ni yedhe ma gayo kute mag ayaki ma imwonyo gi miyo gi timo nyathine nadi?
2. **Pimo thieth mar Option B+ kod thieth mar geng’o landruok kute mag ayaki ka wuok kuom miyo ka dhi ne nyathine**
   1. Gin ang’o ma isewinjo/neno kalure gi thuolo ma mine man e oganda u tiyo kod yedhe mag kute mag ayaki mondo gigeng’ lando kute mag ayaki ne nyithindgi mayom ? Giweyo mwonyo yedhe gi e thuolo mane?
   2. Bende isewinjo wach kuom yiero moro ma miyo mapek man kod kute mag ayaki chako mwonyo yath ne ngimane owuon e kinde ma oyach kendo odhi nyime gi mwonyo yedhe go e ngimane duto ? Yiero ni iluongo ni Option B Plus. Yiero ni dwoko kwan mag kute mag ayaki man e dend miyo kendo rito ngimane, to e seche go bende ogeng’o lando kute mag ayaki ne nyathine mayom.
      1. Ang’o ma iparo kuom yiero no? Ang’o ma nyalo bedo ber mage? Ang’o ma nyalo bedo rach mage?
      2. Kata, bende inyalo hero mondo jaodi/jahera ni ma dhako omwony mana yath kende e kinde ma oyach kod ka odhodho kendo owe bang’ tieko dhodho nyathi ? Nang’o?
      3. Iparo ni jaodi/jahera ni ma dhako nyalo yiero ang’o ? Gin ang’o ma omiyo onyalo timo kamano?
   3. In gi paro mage kuom jaodi/jahera ni ma dhako mwonyo yedhe ma gayo kute mag ayaki e ngimane duto , kata ka obedo ni podi ok owinj ka otuo?
   4. Bende iparo ni mine mapek e oganda u nyalo rwako Option B+? Nang’o kata nang’o ok ginyal? To in ? Gin ang’o ma imiyo ibedo gi yiero no?

**C. Gik ma mono kod ma konyo mwonyo yedhe ma gayo kute mag ayaki kaka dwarore e ngima ng’ato duto**

1. Gin pek mage mabet kuom mwonyo yedhe ma gayo kute mag ayaki (ART) pile ka pile?
2. Bende ji nyisoga monde gi/chuo gi/johera gi ni gimwonyo yedhe ma gayo kute mag ayaki ? Nang’o kata nang’o ok gitim kamano? To jok mamoko man e familia? To oganda?
3. Gin pek mage makende ma jomachuo bedo god kuom mwonyo yedhe gi ma gayo kute mag ayaki (ART)?
4. Gin pek mage ma kende ma jomamine mapek kod mosenyuol bedo godo kuom mwonyo yedhe gi ma gayo kute mag ayaki pile ka pile?
5. Gin gik mage ma iparo ni nyalo konyo ji mondo omwony yedhe gi ma gayo kute mag ayaki (ART) pile ka pile?
6. If the man has/is taking ART:
7. Gin pek mage ma in iwuon isebedo godo/ ma in godo kuom mwonyo yedhe ma gayo kute mag ayaki ?
8. Bende nitie odiochienge ma osekalo ma ok imwonyo yedhe gi mag kute mag ayaki? Nyisa kuom achiel kuom odiochienge go kod gima ne otimore? Ang’o ma ne omiyo/gik mage mane omiyo ok imwonyo yedhe go ? Ang’o mane otimore nikech mano?

**D. Gik ma mono kod ma konyo e bedo kaka dwarore e rit mar kute mag ayaki**

1. Iparo ni en gimaduong moromo nadi mondo ji odhi kaka dwarore e klinik mar kute mag ayaki ne limbe ? Gin pek mage ma ji bedo godo e dhi e limbe mag klinik mar kute mag ayaki?
2. Gin pek mage makende ma jomachuo bedo godo e biro ne limbe mag klinik mar kute ayaki?
3. Gin pek mage makende ma jomamine mapek kod ma osenyuol bedo godo e biro e limbe mag klinik mar kute mag ayaki?
4. Gin gik mage ma nyalo konyo ji mondo obi e klinik ne limbe mag klinik mar kute mag ayaki?
5. If the man is an HIV clinic patient:
6. Gin pek mage ma in go kuom biro e klinik ne limbe mag klinik mar kute mag ayaki?
7. Ibiro ka moluwore marom nade e klinik mar kute mag ayaki? Gik kit kony mage ma iyudo ga e thuolo ma ibiro e klinik no?
8. Yie inyisa kuom bedo mari e kony mamoko mag thieth kata grube ma ji siro go jowete (support group) gi e oganda ma idak e.
9. Bende seche moko isebedo ma ok inyal dhi e limbe mar klinik mar kute mar ayaki ma ochan? Bende inyalo nyisa kuom achiel kuom mago kod gima ne otimore?
10. Bende jatij thieth osegalimi e odi ka ilewo ne limbe mar klinik mar kute mag ayaki? Ne iwinjo nadi kuom limbe no?
11. Ineno nadi kuom biro e klinik bang’ ka iselewo ne limbe ?
12. Gin ang’o ma jotij thieth nyalo timo e klinik mondo okonyi mwonyo yedhe gi kendo odwog e limbe mochan?

**E. Rwako mine man e oganda ma tiego jomamoko**

Dwaher mar nyisi kuom chenro ma iluongo ni “community Mentor Mothers”. Mine ma tiego ji ibiro yier kuom jomamine man e oganda u kendo ma odak kod kute mag ayaki. Gibiro timo limbe ma dala ne jomamine man kod kute mag ayaki gi chuo gi/johera gi machuo e oganda gi mondo gikony e yangruok e yo maber, siro yor pidho nyathi ma ok kete e thuolo mar gamo kute, medo siro bedo e achiel gi rageng kod komo nyuol, jiwo ji mondo opim nythindgi mayom chon kendo timo ne ji limbe, kod jiwo mwonyo yedhe ma gayo kute mag ayaki kaka dwarore kod dwogo e limbe mag rit mar kute mag ayaki .

1. Iparo nade kuom chenro ni?
2. Bende isegatudori kod mine matiego ji e klinik mar jomamine gi nyithindo? Yie inyisa kaka ne iwinjo kata gima ne ineno.
3. In gi paro mage kuom mine ma tiego ji (mentor mothers) bedo e oganda, kar bedo e kar thieth ma gibedo ga?
4. Iparo ni ji biro winjo nade kuom bedo ni jomamine ma tiego ji tiyo e oganda u?
5. Gin kido mage ma jomamine ma tiego ji gi onego obed godo?
6. Gin ang’o ma jomamine ma tiego ji gi onego ong’I ahinya ka gitiyo e oganda?

**F. Rwako kod wach ma onego obed e ote machwok mar sime motudore kod mwonyo yath kaka dwarore kendo keto ji dhi e limbe kaka dwarore**

Dwaher mar nyisi kuom chenro ma iluongo ni “mobile phone text messaging”. E chenro ni mine biro yudo ote machwok mar sime e simbe gi mag ong’we yamo mondo giyud weche kuom ngima familia kendo paro ne gi ni onego gimwony yath kod limbe mag klinik ne gin kod nyithindgi. Ka gidwaro, wuon nyathi bende nyalo yudo ote machwok no bende. Jomamine gi jomachuo bende nyalo oro ote machwok maonge chudo ne jatij thieth, mar biro goyo ne gi sime mondo onyis gi gik ma gidwaro ng’eyo kendo dwoko penjo moro amora.

1. Iparo nade kuom chenro ni?
2. Bende in gi simb ong’we yamo ma mari? Ka ok kamano, bende nitie sime ma uriwo gi ng’ato? Bende jaodi/jahera ni ma dhako nigi simb ong’we yamo ma mare ?
3. Iparo nadi kuom yudo ote machwok kuom ngima familia e simb ongw’e yamo ni (mobail)? To ote machwok kuom yedhe mag kute mag ayaki, limbe mag klinik mar kute mag ayaki, kod pimo nyithindo mayom ne kute mag ayaki?
4. Gin thor weche mage ma moko ma iparo ni onego omedi e ote machwok?
5. Bende inyalo dwaro yudo ote machwok gi e sime ma uriwo? Gin kit ote machwok mage ma ji nyalo rwako mondo oyud e sime ma uriwo?
6. Iparo nadi kuom wuone/johera machuo bende yudo ote machwok mchal kama? Bende kit ote moko machwok beyo moloyo ni wuone/johera machuo ?
7. Iparo nadi kuom gima jomachuo mamoko ma ing’eyo nyalo neno ka giyudo ote machwok machal kamago?
8. Gin ang’o ma onego watang’ne ahinya ka waloso kendo oro ote machwok gi?kuom ranyisi, bende nitiere thor weche moko kata weche ma onego kik tigo?
9. Iparo ni ote machwok mag sime gi onego obi maluwore marom nadi kendo ginyalo konyo ka gibiro moluwore machal nadi? (Pile pile? Dichiel e juma? Dichiel e dwe?)Gin seche mage e odiochieng’ ma_diher mar yudo ote machuok?
10. Bende inyalo konyo yudo ote machwok gi bang’ ka nyathi osenyuol? Kuom thuolo maromo nadi?
11. Gin thor weche mage monego ote machwok mioroni twagie bang’ ka nyathi osenyuol
12. **Daher mar somoni moko kuom ote machwok ma waparo mar oro ne mine kaka in bang ka nyathi osenyuol. Kuom moro ka moro abiro penji gima iwinjo/fuono e ote machwok no,bende ibiro yie mar yudo ote machwok gi e simbi mar ongwe yamo, bende ote machwok gi biro bedo gi kony, kod paro moro amora ma dibedgo e keto makare ote machwok gi.**

- Amosi (name) wigni akwayi ni ikel nyathi ma(name) e klinik e chanjo mondo ogeng’ tuoché mayudo nyithindo kapod kithindo kendo ma biro miyo nyathi ma(name) dong makare kendo ma otegno, bende ibiro bedo gi twak mar hocho kuom rito nyathi ma(nyako/wuoyi) e yo makare
- Amosi (name) Itimo maber kuom limo klinik wigni. Asayi ni gochi kata e flash XXXXXXXX ka in kod penjo kuom ngima ni kata gima mar nyathini. Wantie ka mar konyi.
- Amosi (name) bed ka ing’eyo ni ngima mar miyo en gima ogen kod gima mar nyathi kaachiel gi familia duto. Asayi mondo ibi e klinik ne neno ma kinde ka kinde ne in iwuon kendo igoch ni XXXXXXXX ka in kod penjo moro amora kuom gima ni.

**III. CLOSING**

Ero kamano kuom thuolo ni. Dwoko gi biro konyo ahinya kuom keto ngima familia ma Kenya maber.

(Correct any important misconceptions and provide referrals to PMTCT or ARV services, if appropriate.)

## APPENDIX C: Focus group discussion guide health care workers/managers_English

**For service providers (health professionals, lay health workers, community mobilizers, and community leaders)**

INTRODUCTION

Nyinga en. .Atiyo gi Kar Nonro mar weche Thieth ma Kenya (KEMRI), Mbalariany mar Kolorado, Denver, kod Mbalariany ma Alabama mantie Birmingham man e piny America e chenro ma dwaro keto maber kony mag thieth mag kute mag ayaki e oganda man e Kenya. Dwaher mar wuoyo kodi kuom pachi kuom geng’o landruok mag kute mag ayaki ne nyithindo ka wuok kuom mine gi, thieth mar kute mag ayaki ne jomamine , kod chenro moko manyien ma konyo mine mapek kod anyuola gi mondo odag ngima maber maonge touché. Gimoro amora ma iwacho e kinde twak ni ok bi nyis ng’ato ibiro ket gi e yor mopondo. Weche ma ichiwo ibiro ti godo e tego kendo keto maber chenro mag thieth.

Onego ing’e ni, Ok ochuno ni nyaka iwuo kuom gima ok idwar wuoyo e, kendo inyalo wedyo twak ni e saa asaya. Twak I biro kawo madirom saa achiel gin us. Ka in gi penjo ma diher penjo kuom weche mamoko, anyalo konyo yudo dwoko bang’ ka twak oserumo.

**(Go through the information sheet for forcus group discussion out loud)** and give participant a copy. Ask permission, and if they agree, start the tape recorder AFTER the introductions part of the discussion. This guide includes the topics to be covered and questions that may be helpful in facilitating the forcus group discussion.You do NOT have to ask all question or follow the order given in the guide . major topic areas and questions are indicated.)

**II. INTRODUCTIONS**

Mokwongo we wa ng’ere. Wadhi aluora mondo ng’ato ka ng’ato ofulre. Inyalo nyiso wa nyingi mokwongo ( kata nying mi gombo tiyo go e twagni ), in ja thieth mane, kendo gimoramora kuomi madi gomb nyiso ji e twagni. ( jo nonro ni bende onego ofulre. Ka jog rube oyie mondo omak dwond gi, inyalo chako mako dwond gi, inyalo chako mako dwond gi bang’ thuolo mar twak.

III. DISCUSSION TOPIC

**8. Pach Oganda kuom yedhe ma itiyo go e gayo kute mag ayaki kod mageng’o landruok kute mag ayaki ka owuok kuom miyo ka dhi ne nyathine.**

- 1. Ere kaka ji e oganda u neno thieth mar kute mar ayaki? Bende gin gi geno ni yedhe ma gayo kute mag ayaki tiyo? Nang’o kata Nang’o ok ipar kamano? Bende nitie kit thieth mamoko ma ji tiyo go e thiedho kute mag ayaki? PROBE for Traditional medicine, witchcraft, etc

1. Ang’o ma Oganda geno kuom kaka mwonyo yedhe ma gayo kute mag ayaki timo jogo ma tiyo kod gi ? Gik ma gitimo mabeyo? Gik ma gitimo maricho?
2. Ere kaka ji e Oganda u neno jogo man e thieth mar kute mag ayaki (ART)?
3. Ere kaka ji e Oganda u winjo kuom miyo mapek ma tiyo kod yedhe ma gayo kute mag ayaki mondo ogen’g landruok mag kute mag ayaki kowuok kuom miyo ne nyathine?

**9. Pimo thieth mar Option B+ kod thieth mar geng’olandruok kute mag ayaki ka wuok kuom miyo ka dhi ne nyathine.**

1. Bende isewinjo wach kuom yiero moro ma miyo mapek man kod kute mag ayaki chako mwonyo yath ne ngimane owuon e kinde ma oyach kendo odhi nyime gi mwonyo yedhe go e ngimane duto? Yiero ni iluongo ni Option B plus. Yiero ni dwoko kwan mag kute mag ayaki man e dend miyo kendo rito ngimane , to e seche go bende ogeng’o lando kute mag ayaki ni nyathine mayom.
2. Ang’o ma iparo kuom yiero no? Ang’o ma nyalo bedo ber mage? Ang;o ma nyalo bedo racch mage?
3. Kata, Bende iparo ni jomamine e oganda u nyalo winjo kuomyiero muonyo yedhe sama gin gi ich gi dhodho kendo weyo kawo thieth bang’ nyuol? Nang’o?
4. In gi paro mage kuom jaodi/ jahera ni machuo manyalo yiero? Nang’o? jo anyuola moko?
5. Bende iparo ni mine mapek e oganda u nyalo rwako Option B+? nang’o kata nang’ ok ginyal? ere kaka inyalo lande e oganda u?
6. bende iparo ni mine mapek e oganda u nyalo rwako Option B+? nang’o kata nang’o ok ginyal? gin ang’O ma imiyo ibedo gi yiero no?
7. iparo ni miyo mapek man gi kute mag ayaki nyalo yiero ang’o ? gin ang’o ma onyalo timo kamano? Bende in gi penjo mora mora kuom option B+?
8. gin pek mane manyalo a e Option B+ Ki tiyo kod gi e yor thieth? Gin pek mane ma unyalo bedo godo kutiyo gi Option B+ kar thieth? Pogo yedhe? Lalo luwo? Ng’iyo gima jotwo e kende mathoth? Gi mamoko?

**C. Gik ma mono kod ma konyo mwonyo yedhe ma gayo kute mag ayaki kaka dwarore e ngima ng’ato duto.**

**10.**chandruok mage ma ji neno e gweng’u e sama gi muonyo yethe mag gao kute mag ayaki( ART) Pilepile?

11. Bende ji nyisoga monde gi/chuo gi/johera gi ni gimwonyo yedhe gi magao kute mag ayaki? Nango kata nango ok gitim kamano? To jok mamoko man e anyuola? To oganda?

12. Gin pek mage makende ma jomamine mapek gi mosenyuol bedo godo kuom muonyo yethe gi ma gayo kute mag ayaki (ART)?

13. Gin gik mage ma iparo ni nyalo konyo jomamine mapek gi mosenyuol mondo omwony yedhe gi ma gayo kute mag ayaki (ART) Pilepile.

**D. Gik ma mono kod ma konyo e bedo kaka dwarore e rit mar kute mag ayaki.**

19. bende jogi mithietho man gi kute mag ayaki, paro ni ber bire e limbe mag klinik mar kute mag ayaki?

Gin pek mage ma ji e ngwengu ni nenoga e biro ne limbe mag klinik mar kute ayaki?

20. Yie inyisa kuom kony mamoko mag thieth kata grube ma ji siro go jowete (support group) mantie e ngwengu ka?

21. bende jotij thieth limo ji ka gilewo ne limbe mar klinik mar kute mag ayaki? Limbe go tiyo nade? Gin pek mage ma in go? Ineno nade kuom biro e klinik ban’g ka jatuo oselewo ne limbe.?

22. Gin pek mage makende ma jomamine mapek kod ma osenyuol bedo godo e biro e limbe mag klinik mar kute mag ayaki?

23. Gin gik mage ma nyalo konyo jomamine mapek kod ma osenyuol mondo obi e klinik ne limbe mag klinik mar kute marg ayaki?

24. Gin ang’o ma jotij thieth nyalo timo mondo okony jotuo mwonyo yethe gi kendo odwog e limbe mochan.

**E. Rwako mine man e oganda ma tiego jomamoko.**

**Dwaher mar nyisi kuom chenro ma iluongo ni “ community Mentor Mothers”. Mine ma tiego ji ibiro yier kuom jomanine man e oganda u kendo ma odak kod kute mag ayaki. Gibiro timo limbe ma dala ne jomamine man kod kute mag ayaki gi chuo gi/ johera gi machuo e oganda gi, mondo gikony e yangruok e yo maber, siro yor pitho nyathi ma ok kete, e thuolo mar gamokute mag ayaki, medo siro bedo e achiel gi rageng kod komo nyuol, jiwo ji mondo opim nyithindgi mayom chon kendo timo ne ji limbe, kod jiwo mwonyo yedhe ma gayo kute mag ayaki kaka dwarore kod dwogo e limbe mag rit mar kute mag ayaki.**

13. Iparo nade kuom chenro ni?

14.bende nitie mine matiego ji e klinik mar jomamine gi nyithindo.

15.In gi paro mage kuom mine ma tiego ji (MENTOR MOTHErs) bedo e oganda, kar bedo e kar thieth ma gibedo ga?

16. iparo ni ji biro winjo nade kuom bedo gi jomamine ma tiego ji (mentor mothers) tiyo e ogandau?

17. gin kido mage ma jomamine ma tiego ji onego obed godo?

18. Gin ang’o ma jomamine ma tiego ji gi onego ong’I ahinya ka gitiyo e oganda?

**F. Rwako kod wach ma onego obed e ote machwok mar sime motudore kod mwonyo yath kaka dwarore kendo keto ji dhi e limbe kaka dwarore.**

Dwaher mar nyisi kuom chenro ma iluongo ni “mobile text messaging”. E Chenro ni, mine biro yudo ote machwok mar sime e simbe gi mag ong’we yamo mondo giyud weche kuom ngima anyuola kendo paro ne gi ni, onego gimwony yath kod limbe mag klinik ne gin kod nyithindgi. Ka gidwaro, wuon nyathi bende nyalo yudo ote machwok no bende. Jomamine gi jomachuo bende nyalo oro ote machwok maonge chudo ne jatich thieth, mar biro goyo ne gi sime mondo onyis gi gik ma gidwaro ng’eyo kendo dwoko penjo moro amora.

1. Iparo nade kuom chenro ni?
2. Bende jomamine e oganda u, ni gi simo on’gwe yamo ma margi” ka ok kamano, bende nitie sime ma giriwo gi ng’ato? Inyalo wacho ni ji adi ma riwo gi gi simb ong’we yamo?
3. Iparo ni jomamine biro yie yudo ote machwok kuom ngima familia e simb on’gwe yamo gi(mobail)? To ote machwok kuom yedhe mag kute mag ayaki, limbe mag klinik mar kute mag ayaki, kod pimo nyithindo mayom ne kute mag ayaki?
4. Gin thor weche mage ma moko iparo ni onego omedi e ote machwok?
5. Bende inyalo dwaro yudo ote machwok gi e sime ma uriwo? Gin kit ote machwok mage ma ji nyalo rwako mondo oyud e sime ma uriwo?
6. Iparo nade kuom wuone/ johera machuo bende yudo ote machwok go? Bende kit ote moko machwok beyo moloyo, ne wuone/johera machuo?
7. Gin ang’o ma onego watan’ne ahinya ka waloso kendo oro ote machwok gi? – kuom ranyisi, bende nitiere thor weche moko kata weche ma onego kik ti go?
8. Di dii ma ote machwok mag sime gi onego obi maluwore marom nadi kendo ginalo konyo ka gibiro moluwore machal nade?( Pile pile, dichiel e juma, dichiel e dwe?) Gin seche mage e odiochieng ‘ ma diher mar yudo ote machuok?
9. Bende nyalo konyo ka jomamine gi jomachuo yudo ote machwok gi bang’ ka nyathi osenyuol? Kuom thuolo ma room nade?
10. Gin thor weche mage mihero monego ote machwok mioroni twagie bang’ ka nyathi osenyuol?

**Daher mar somoni moko kuom ote machwok ma waparo mar oro ne mine gi wuon nyathi bang’ ka nyathi osenyuol. Kuom moro ka mora abiro penji gima iwinjo/fuono e ote machwok no. bende kaka ibiro yie mar yudo ote machwok gi simbi mar ong’we yamo, bende ote machwok gi biro bedo gi kony, kod paro moro amora ma dibedgo e keto makare ote machwok gi.**

- - Mar jomamine gi wuone: Amosi (nying) e juma ni akwayi ni ikel nyathi (nying nyathi) e klinik e chanjo mondo ogeng’ touché mayudo nyithindo kapod gi thindo kendo mabiro miyo nyathi ma (nying nyathi) dong makare kendo ma otegno. Bende ibiro bedo gi twak mar hocho kuom rito nyathi ma (nyako/wuoyi) e yo makare.
  - Mar jomanine gi wuone: Amosi (nying) Itimo ma ber kuom limo klinik e juma ni. Asayi ni gochi kata igo sime to ing’adi mondo ogoch ni. Ka in kod penjo kuom ngima ni kata gima mar nyathini. Wantie ka mar konyi.
  - Mar jomamine kende: Amosi (nying) bed ka ing’eyo ni ngima mar miyo en gima ma ogen kod ngima mar nyathi kaachiel gi familia duto. Yie ibi e klinik ne neno ma kinde ka kinde ne in iwuon kendo igochi kata igo sime to ing’ad mondo ogoch ni XXXXXXXXXXXX Ka in kod penjo moro amora kom gima ni
  - Mar jomachwo kende: Amosi (nying) bed ka ing’eyo ni ngima mar wuon en gima ma ogen kod ngima mar nyathi kaachiel gi familia duto. Yie ibi e klinik ne nene ma kinde ka kinde ne in iwuon kendo igochi kata igo sime to ing’adi mondo ogoch ni XXXXXXXXXXXXXX Ka in kod penjo moro amora kuom gima ni.
  - Gin thor weche mage ma moko ma iparo ni onego omedi e ote machwok ne jomamine( gi nyithindo gi) kod mwonyo yath mar geng’o landruok kute mag ayaki kaka dwarore kendo dhi e limbe kaka dwarore?

**G. Other suggestions**

In gi paro mage maber kuom gima inyalo medi ne pimo thieth mar Option B+ E Oganda u / kar thieth.

**IV. CLOSING**

Ero kamano kuom thuolo ni. Dwoko gi biro konyo ahinya kuom keto ngima familia ma Kenya maber.
